# Clusters of lineage-specific genes are anchored by ZNF274 in repressive perinucleolar compartments

**DOI:** 10.1126/sciadv.ado1662

**Published:** 2024-09-13

**Authors:** Martina Begnis, Julien Duc, Sandra Offner, Delphine Grun, Shaoline Sheppard, Olga Rosspopoff, Didier Trono

**Affiliations:** School of Life Sciences, École Polytechnique Fédérale de Lausanne, Lausanne, Switzerland.

## Abstract

Long known as the site of ribosome biogenesis, the nucleolus is increasingly recognized for its role in shaping three-dimensional (3D) genome organization. Still, the mechanisms governing the targeting of selected regions of the genome to nucleolus-associated domains (NADs) remain enigmatic. Here, we reveal the essential role of ZNF274, a SCAN-bearing member of the Krüppel-associated box (KRAB)–containing zinc finger protein (KZFP) family, in sequestering lineage-specific gene clusters within NADs. Ablation of ZNF274 triggers transcriptional activation across entire genomic neighborhoods—encompassing, among others, protocadherin and KZFP-encoding genes—with loss of repressive chromatin marks, altered the 3D genome architecture and de novo CTCF binding. Mechanistically, ZNF274 anchors target DNA sequences at the nucleolus and facilitates their compartmentalization via a previously uncharted function of the SCAN domain. Our findings illuminate the mechanisms underlying NAD organization and suggest that perinucleolar entrapment into repressive hubs constrains the activation of tandemly arrayed genes to enable selective expression and modulate cell differentiation programs during development.

## Teaser

ZNF274 tethers tandemly arrayed genes to NADs, thus inducing their silencing and enabling selective promoter activation.

## INTRODUCTION

The eukaryotic genome is spatially organized to guarantee the orderly execution of transcriptional programs (*1*, *2*). With hundreds of genes switching between active and inactive compartments during development and differentiation (*3*, *4*), nuclear bodies can serve as a scaffold for this reorganization (*5*, *6*). In most nuclei, heterochromatin gathers at the nuclear lamina or around the nucleolus. While lamina-associated domains (LADs) are extensively mapped, the membraneless nature of the nucleolus has hampered the identification of their nucleolus-associated domain (NAD) counterparts (*7*, *8*). Thus, what is the role of perinucleolar heterochromatin in genome organization and what directs specific gene subsets to this region remain unanswered questions.

The nucleolus is the largest identified nuclear substructure and is best known as the site of ribosome biogenesis. Silent ribosomal DNA (rDNA) repeats condense at the nucleolar periphery together with centromeres and pericentromeric regions. Early investigations into human NADs using biochemical purification of nucleoli revealed an enrichment of specific gene subsets, including protocadherins (PCDHs), immunoglobulins, and olfactory receptors, which are all characterized by their organization in gene clusters, heterochromatinization, and cell type–restricted expression (*7*, *9*–*12*). These findings led to the hypothesis that the nucleolus may act as a specialized compartment for establishing repressive chromatin and regulating dynamic lineage-specific expression programs.

Unraveling the determinants of NADs is critical to shed light on the role of the nucleolus in broad epigenetic regulatory mechanisms and comprehend how nuclear compartments can affect gene expression patterns in development and disease. While a few proteins have been implicated in assisting chromatin interactions with the nucleolus (*13*–*17*), if they truly serve as direct anchors was not established. Among the gene clusters enriched at NADs are *KZFPs*, responsible for transcription factors encoded in hundreds by the human genome (*15*). *KZFP* genes are decorated with a unique H3K9me3 chromatin signature deposited over their 3′ end by one of their family members, ZNF274, via the Krüppel-associated box (KRAB)–mediated recruitment of KAP1/TRIM28 and associated SETDB1 (*16*). Notably, ZNF274 was shown to have nucleolar targeting ability and computational modeling found it to be a topologically associating domains (TAD) boundary–associated genomic element (*17*, *18*). Despite these intriguing connections linking ZNF274 to the perinucleolar environment, its role in three-dimensional (3D) chromatin architecture and gene regulation has thus far remained undetermined. The present study reveals that it is responsible for targeting clusters of lineage-specific genes to repressive perinucleolar subdomains.

## RESULTS

### ZNF274 represses lineage-specific genes organized in clusters

**Fig. 1.**
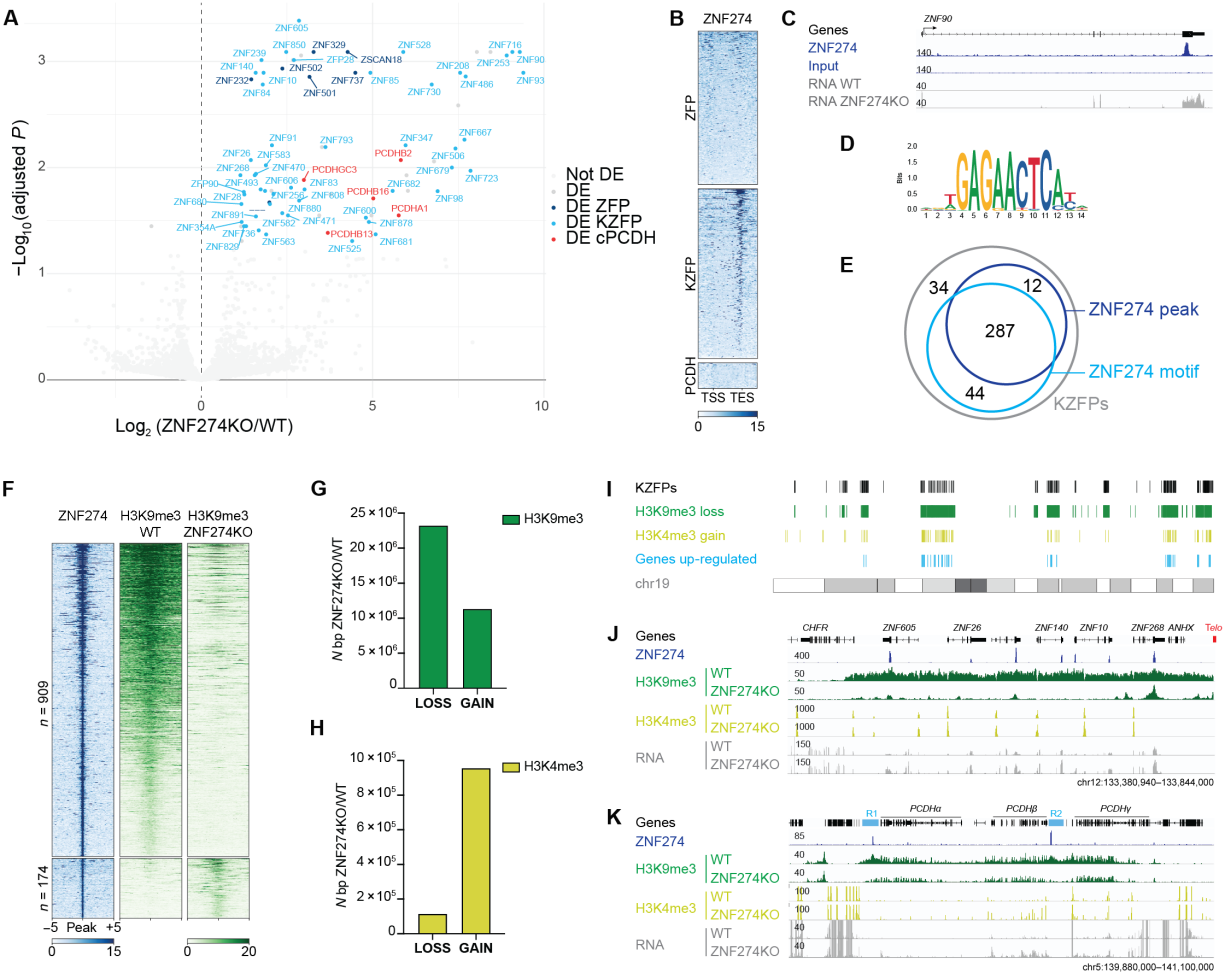
ZNF274 represses lineage-specific genes organized in clusters. (**A**) Volcano plot comparing the FC in gene expression for WT and *ZNF274* KO HEK293T cells. Representative *ZFP*, *KZFP*, and *PCDH* genes are highlighted in blue, light blue, and red, respectively. DE, differentially expressed. (**B**) Heatmap of ZNF274 ChIP-seq enrichment across the body of *ZFP*, *KZFP*, and *PCDH* genes [+2 kb from the transcription start site (TSS) or transcription end site (TES)]. (**C**) Integrative Genomics Viewer (IGV) browser screenshot of a representative *KZFP* gene showing tracks for ZNF274 ChIP-seq in *ZNF274* KO cells overexpressing HA-tagged ZNF274 and RNA-seq in WT and *ZNF274* KO cells. (**D**) Top-scoring DNA binding motif identified for ZNF274. (**E**) Venn diagram showing the overlap of *KZFP* genes harboring the ZNF274 motif and ChIP-seq peaks. (**F**) Heatmap of ZNF274 and H3K9me3 ChIP-seq across ZNF274 peaks showing the differential enrichment of H3K9me3. Each row represents a 5-kb window centered on peak midpoint, sorted by the ZNF274 ChIP signal. (**G**) Bar plot depicting significant loss or gain of H3K9me3 in WT versus *ZNF274* KO HEK293T cells (FC > 2; adjusted *P* value < 0.05; *n* = 4 replicates per group). (**H**) Bar plot depicting significant loss or gain of H3K4me3 in WT versus *ZNF274* KO HEK293T cells (FC > 2; adjusted *P* value <0.05; *n* = 2 replicates per group). (**I**) Karyotype plot of chromosome 19 showing the genomic localization of regions significantly affected upon *ZNF274* KO. (**J**) IGV browser screenshots showing tracks for ZNF274 ChIP-seq in *ZNF274* KO cells overexpressing HA-tagged ZNF274, H3K9me3, and H3K4me3 ChIP-seq in WT and *ZNF274* KO cells across the subtelomeric ZFP cluster. The red box indicates the position of telomeric repeats. (**K**) IGV browser screenshots showing tracks for ZNF274 ChIP-seq in *ZNF274* KO cells overexpressing HA-tagged ZNF274, H3K9me3, and H3K4me3 ChIP-seq in WT and *ZNF274* KO cells across PCDH. “R1” and “R2” indicate the tracks of H3K9me3 chromatin undergoing substantial shrinkage after ZNF274 loss.

To analyze the biological function of ZNF274, we generated homozygous knockout (KO) human embryonic kidney (HEK) 293T cells by CRISPR-Cas9 genome editing (fig. S1, A and B). Transcriptome profiling by RNA sequencing (RNA-seq) revealed that almost all (79/80) protein-coding genes with significantly altered mRNA levels in *ZNF274* KO cells were up-regulated (Fig. 1A and table S1). Sixty of them encoded KRAB-containing zinc finger proteins (KZFPs) and eight for some other C_2_H_2_ zinc finger proteins (ZFPs). Standing out among transcripts up-regulated in the absence of ZNF274 were also products of the PCDH gene family. PCDHs are adhesion molecules predominantly expressed in the nervous system and, like KZFPs, are encoded by genes organized into tandem arrays (α, β, and γ clusters on chromosome 5). We found representatives from all *PCDH* clusters to be significantly more transcribed in *ZNF274* KO cells (Fig. 1A). We were struck by the similarities of genomic modularity and regulatory logic displayed by *KZFP* and *PCDH* genes, for which the ground state of most promoters is repressed. Nevertheless, previous profiling of ZNF274 genome-wide binding sites did not reveal an enrichment at promoters of genes deregulated in our KO mutants (*15*). To explore the issue further, we reintroduced hemagglutinin (HA)–tagged ZNF274 in KO cells and performed chromatin immunoprecipitation sequencing (ChIP-seq) (fig. S1C). Of over 20,000 significantly enriched sites, most were located in introns or intergenic regions (fig. S1, D and E, and table S2). We found that ZNF274 especially accumulated over the 3′ end of *KZFP* genes but not on other *ZFP* or *PCDH* genes (Fig. 1, B and C, and fig. S1F). Motif analysis of ZNF274-associated loci confirmed that the top-scoring motif for ZNF274 (Fig. 1D) was present in the zinc finger (ZNF)-encoding exons of 331 out of 377 *KZFP* genes (Fig. 1E). ZNF274 peaks are ingrained in heterochromatin marked by H3K9me3, and these loci failed at maintaining the silencing mark upon ZNF274 depletion (Fig. 1F). Loss of H3K9me3 was not restricted to ZNF274 peaks but extended across large portions of the genome including regions located in pericentromeric and subtelomeric domains, where lack of repressive histone modifications was concomitant with an increase in the H3K4me3 activating mark (Fig. 1, G to I). Prominent alterations in chromatin marks were identified within segments housing *KZFP* gene clusters (Fig. 1J and fig. S2A). At the *PCDH* locus, two ZNF274-recruiting regions (R1 and R2) interspersed between *PCDH* clusters underwent substantial shrinkage of H3K9me3-adorned chromatin after ZNF274 loss (Fig. 1K). On the contrary, a minority of sequences displayed a gain of H3K9me3 and were enriched in binding sites for other KZFPs, mirroring the functional activation of these repressors (fig. S2, B to D). Given the profound cross-talk between chromatin modifications and DNA methylation, we used the enzymatic methyl sequencing (EM-seq) method to interrogate the impact of the large-scale H3K9me3 contraction on CpG methylation (mCG). We detected only a minor decline in mCG on the promoter of *KZFP* genes, which are mainly marked by unmethylated DNA in HEK293T cells (fig. S3A). On the other hand, many of the H3K9me3 domains affected by loss of ZNF274 showed an overall increase in mCG level (fig. S3, B and C). The deposition of mCG was particularly pronounced on *KZFP* and *PCDH* gene bodies, where it correlated with the concurrent accrual of H3K36me3 histone mark, known to recruit the de novo methyltransferase DNMT3b (fig. S3, D to F) (*19*). Likewise, DNA segments harboring ZNF274 peaks displayed increased mCG levels specifically where depletion of the KZFP led to loss of H3K9me3 (fig. S3G).

**Fig. 2.**
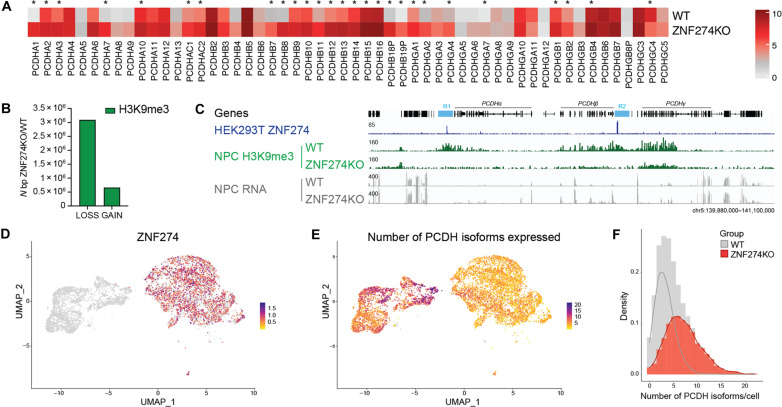
ZNF274 is required for selective and combinatorial expression of *PCDH* genes. (**A**) Expressed *PCDH* genes in WT versus *ZNF274* KO NPCs. Data are shown as the average of *n* = 2 parental cell lines. Asterisks indicate genes whose expression significantly changes in WT versus *ZNF274* KO NPCs. (**B**) Bar plot depicting significant loss or gain of H3K9me3 in WT versus *ZNF274* KO NPCs (FC > 2; adjusted *P* value < 0.05; *n* = 4 replicates per group). (**C**) IGV browser screenshot showing tracks for HA-tagged ZNF274 in HEK293T cells, H3K9me3 Cut&Tag and RNA-seq in WT and *ZNF274* KO NPCs across the *PCDH* gene cluster. (**D**) UMAP visualization of WT and *ZNF274* KO NPCs obtained through 10X 5′-end single-cell sequencing. The UMAP is colored by the expression of *ZNF274* gene. (**E**) The same UMAP as in (A), colored by the expression of the number of *PCDH* genes. (**F**) Density plot representing the distribution of cells expressing varying numbers of PCDH isoforms under either WT or *ZNF274* KO condition. The superimposed curves represent kernel density estimate for each condition.

The data presented thus far suggest that ZNF274-mediated heterochromatin formation is the main mechanism securing robust transcriptional silencing of *KZFP* and *PCDH* gene clusters. Because of the neuron-specific expression of *PCDHs*, we decided to confirm the functional role of ZNF274 in regulating these genes in human neural progenitor cells (NPCs) derived from embryonic stem cells (ESCs) (fig. S4, A and B). Genes encoding 28 of 56 isoforms of PCDH were up-regulated in NPC cultures generated from homozygous *ZNF274* KO ESCs (Fig. 2A and table S3). We also observed the up-regulation of most *KZFP* genes in NPCs (fig. S4C) as well as other tandemly arrayed genes that have lineage-specific functions, such as the kallikrein (*KLK*), pregnancy-specific glycoprotein (*PSG*), keratin (*KRT*), and nucleotide-binding oligomerization domain-like receptor protein (*NLRP*) families (table S3). Genes down-regulated in KO cells were particularly enriched for Gene Ontology terms related to neuron differentiation and brain development (fig. S4D), suggesting that ZNF274 plays a critical role for the realization of these processes either directly or via the regulation of other suppressors such as KZFPs. Using Cut&Tag, we verified that H3K9me3 levels were globally reduced in KO NPCs, similar to those observed in HEK293T cells (Fig. 2B and fig. S4E). Notably, the R1 and R2 loci in the *PCDH* locus lost H3K9me3 in KO cells (Fig. 2C), as did all the gene families just listed as up-regulated in this setting (fig. S4, F to H). In particular, *ZNF274* KO caused loss of H3K9me3 on the Prader-Willi syndrome (PWS)–associated *SNORD116* cluster, known to be epigenetically regulated by ZNF274, stressing the particular relevance of ZNF274 function in the neuronal context (fig. S4I) (*20*, *21*). Conversely, the large *HIST1* cluster on chromosome 6 containing 55 histone genes and localizing within transcriptionally active nuclear bodies (histone locus bodies) was unperturbed (fig. S4J) (*22*). We conclude that binding of ZNF274 serves as the pivotal catalyst for the assembly of heterochromatin that permeates genes organized in clusters, thereby thwarting any spurious activation of lineage-restricted expression programs.

### ZNF274 is required for selective and combinatorial expression of tandemly arrayed genes

The combinatorial expression of different PCDH isoforms is crucial for proper dendritic arborization because it provides neurons with a unique barcode to distinguish self from nonself (*23*). While the mechanisms involved in *PCDH* stochastic promoter choice are difficult to study in vivo because each neuron expresses at low levels a distinct repertoire of PCDH alternate exons, we could clearly detect a strong gain of expression of PCDH isoforms in our experiments. To test if individual cells expressed an increased number of PCDH isoforms rather than higher levels of individual ones, we performed single-cell RNA-seq (scRNA-seq) of wild-type (WT) and KO NPCs (Fig. 2D). This revealed that a larger proportion of *PCDH* isoforms were transcribed by each cell upon loss of *ZNF274*, in violation of the “one isoform per neuron” rule that safeguards neuronal self-avoidance (Fig. 2, E and F). In parallel, we found many more *KZFP* genes to be actively transcribed in individual *ZNF274* KO cells (fig. S5, A and B). Control regions such as the *HIST1* cluster on chromosome 16 maintained similar expression levels under WT and KO conditions (fig. S5, C and D), while in contrast, we confirmed the activation of other clustered gene families regulated by ZNF274 (fig. S5, E and F). Collectively, these findings strongly suggest that ZNF274-dependent repressive mechanisms play an essential role in constraining the selective activation of tandemly arrayed genes so that loss of ZNF274 activity precludes the fine-tuning of isoform diversity that allows cells to achieve phenotypic and functional diversification during embryogenesis and differentiation.

### ZNF274 occupancy hampers binding of CTCF and promotes genome compartmentalization

**Fig. 3.**
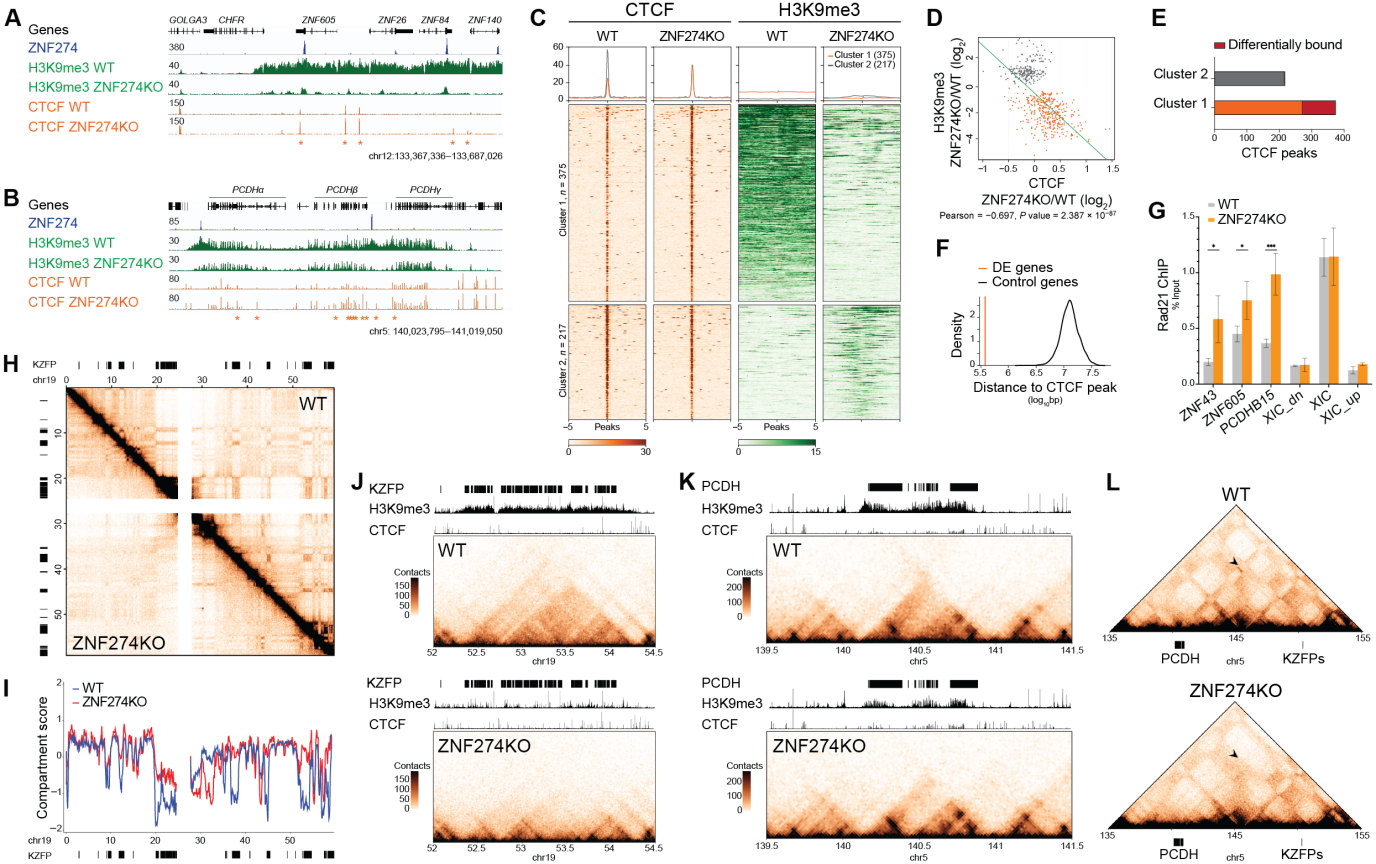
ZNF274 occupancy hampers binding of CTCF and promotes genome compartmentalization. (**A**) IGV browser screenshot showing the ChIP-seq signal of HA-tagged ZNF274, H3K9me3, and CTCF in *ZNF274* KO cells (*, adjusted *P* value < 0.1; FC > 1.5). (**B**) Same as in (A) across PCDH (*, adjusted *P* value < 0.1; FC > 1.5). (**C**) Heatmap and quantification of CTCF and H3K9me3 ChIP-seq enrichment across CTCF peaks lying within hypo- or hypermethylated H3K9me3 regions. (**D**) Scatterplot displaying the inverse correlation between H3K9me3 and CTCF enrichment in WT versus *ZNF274* KO cells. The Pearson correlation coefficient was calculated to measure the relationship between the two variables. (**E**) Bar plots depicting significant loss or gain of CTCF in WT versus *ZNF274* KO HEK293T cells (FC > 1.5; adjusted *P* value < 0.1; *n* = 3 replicates per group). (**F**) Density plot showing the average distance of significantly different CTCF peaks from differentially expressed genes in *ZNF274* KO cells compared to a “random” set of control genes. (**G**) ChIP-qPCR of RAD21 on significantly enriched CTCF peaks in *ZNF274* KO HEK293T cells. XIC, X-inactivation center; XIC_dn and XIC_up are negative controls downstream and upstream of XIC, respectively (*P < 0.05; ***P < 0.001). (**H**) Normalized Hi-C matrix at 100-kb resolution of chromosome 19 displaying contact frequencies in WT (upper triangle) and *ZNF274* KO (lower triangle) HEK293T cells. (**I**) Compartment scores of chromosome 19 revealing changes in A (>0) and B (<0) compartment segregation. (**J**) Normalized Hi-C maps at 20-kb resolution for a *KZFP* gene cluster in WT and *ZNF274* KO HEK293T cells. On the top are tracks visualizing *KZFP* genes (black bars) and the ChIP-seq signal of H3K9me3 and CTCF. (**K**) Same as in (J) for the *PCDH* gene cluster. On the top are tracks visualizing *PCDH* genes (black bars) and the ChIP-seq signal of H3K9me3 and CTCF. (**L**) Pyramid plot at 100-kb resolution showing contacts (black arrow) between PCDH and KZFP.

The replacement of repressive by activating histone marks upon *ZNF274* KO suggested a widespread recruitment of transcription factors altering enhancer-promoter communication. At the *PCDH* locus, it is well documented that transcriptional activation requires the formation of CTCF-driven DNA looping between gene promoters and downstream enhancers (*24*–*27*). Therefore we performed ChIP-seq analysis of CTCF in HEK293T cells. Newly gained CTCF peaks were found on *PCDH* promoters in the absence of ZNF274 (Fig. 3A); likewise, the CTCF signal increased across *KZFP* clusters (Fig. 3B). In general, regions that experienced a reduction in H3K9me3 showed a mirroring rise in CTCF, whereas in areas that gained H3K9me3, CTCF occupancy decreased (Fig. 3, C and D). A total of 99.4% (173/174) of sequences with significantly altered CTCF recruitment represented enhanced (including de novo) peaks (Fig. 3E) and were enriched in the vicinity of ZNF274-controlled genes (Fig. 3F), with 136/174 containing a CTCF motif (JASPAR MA0139.1; *P* value < 0.00005). Because the two proteins often colocalize at CTCF barriers (*28*), we verified that the cohesin subunit RAD21 was also recruited to newly gained CTCF sites in KO cells while remaining unchanged at control regions (Fig. 3G). The mutual exclusivity between ZNF274 and CTCF occupancy led us to investigate if the absence of ZNF274 would perturb chromatin spatial organization. We performed Hi-C for three WT and three KO HEK293T cell clones and pooled the data after verifying the high quality of the triplicates (fig. S6A). Overall chromosomal interaction landscapes were very similar between WT and KO cells, but chromosome 19, which is where, in humans, most *KZFP* clusters are found (*29*), displayed a notable decrease in the number of far-*cis* contacts (>10 Mb) (fig. S7A). Genome partitioning of chromosome 19 into A and B compartments was exceptionally affected in KO cells, indicating that the segregation between *KZFP*-containing compartments was less strict in this setting (Fig. 3, H and I). Other *KZFP* clusters located on different chromosomes showed similar defects; elsewhere, the interaction landscape was unaltered (fig. S7, B to E). Analysis of the Hi-C data revealed a weakening of insulation of the *PCDH* locus, concomitant with the appearance or strengthening of architectural “stripes” along the α, β, and γ clusters (Fig. 3J), a feature that has been associated with the activity of cohesins in the assembly of enhancer-promoter complexes (*30*). Local rewiring of chromatin interactions at the *PCDH* locus was further supported by a series of UMI-4C experiments using contact profiling baits targeting ZNF274 peaks in R1 and R2 as well as the annotated enhancers HS5-1 and HS16 (fig. S8A). UMI-4C data confirmed the changes observed in our Hi-C maps and yielded additional observations. Not only the relative contact strength between enhancers and *PCDH* alternate exons was increased in KO compared to WT cells, but we could also detect a reduction in long-range contacts between R1 and R2 in the absence of ZNF274. R1 and R2 correspond to robust SETDB1-sensitive H3K9me3 peaks that, in mice, have been shown to form repressive loop bundles regulating the *PCDH* locus (*24*). In humans, R1 polymorphism has been associated with an increased risk of schizophrenia (chr5: 140,023,664 to 140,222,664) by the Psychiatric Genomics Consortium (*31*). R1 carries the noncoding single-nucleotide polymorphism (SNP) rs11189671 about 70 bp from the ZNF274 identified motif, whose dosage has been proven to correlate with the expression of multiple *PCDH* genes in the adult frontal cortex (*32*). Using a green fluorescent protein (GFP) reporter system, we confirmed that R1 acts as a ZNF274-dependent *cis* repressor and that the risk SNP rs111896713 partially dampens its influence (fig. S8, B and C). Therefore, ZNF274-controlled R1 and R2 serve as key organizers for local chromosomal conformations at the *PCDH* locus and exert repressive effects on gene expression. Unexpectedly, we found long-range DNA contacts between *PCDH* and two *KZFP* clusters located ~10 and ~40 Mb away, respectively (Fig. 3L and fig. S7F). These contacts, which depend on ZNF274, imply the presence of a shared nuclear subcompartment where these genes potentially segregate for coregulation. The formation of ZNF274-dependent multicluster hubs is a conserved feature across different cell types, with the same type of *trans* interactions between *KZFP* and *PCDH* genes detected in both HEK293T cells and NPCs (figs. S6B and S9, A to D). Together, our data demonstrate that binding of ZNF274 instigates rearrangements in the 3D genome architecture that are tailored to segregate lineage-specific genes into repressive compartments impermeable to CTCF occupancy.

### The SCAN domain is responsible for bringing ZNF274 to the nucleolus and promoting the formation of repressive compartments

**Fig. 4.**
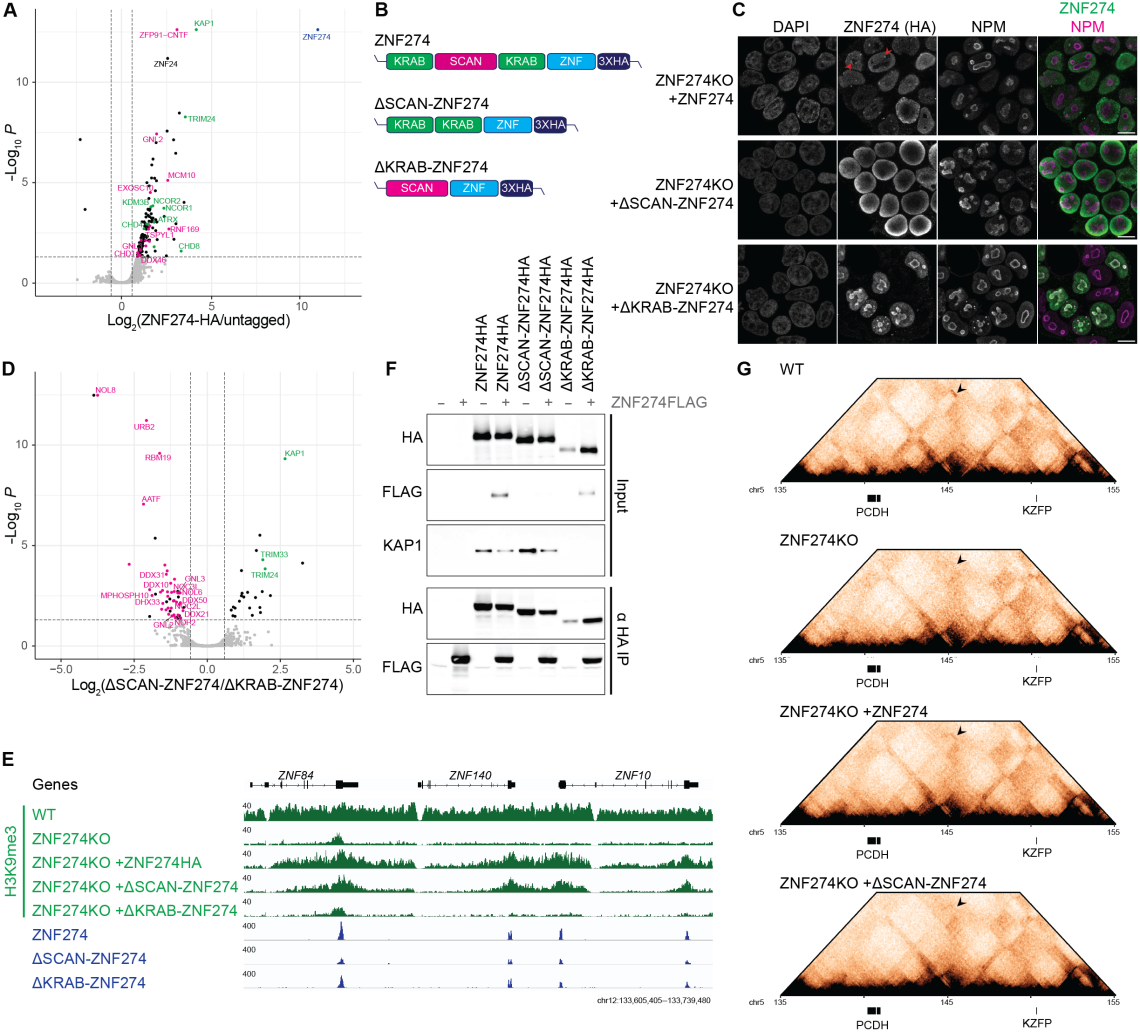
The SCAN domain is responsible for bringing ZNF274 to the nucleolus and promoting the formation of repressive compartments. (**A**) Volcano plot of mass spectrometry analysis of biotinylated proteins after targeting ProtA-TurboID to ZNF274-HA in HEK293T cells (*n* = 3 replicates per condition). A selection of significant interactors are highlighted. Green dots indicate silencer complexes, pink dots indicates proteins known to be localized at nucleoli, and blue dot indicates ZNF274 (bait). (**B**) Schematic representation of ZNF274 domains and relative mutants. (**C**) Representative confocal images of doxycycline-induced ZNF274-HA, ΔKRAB-ZNF274-HA, and ΔSCAN-ZNF274-HA, the nucleolar marker B23 (NPM) protein, and DAPI in *ZNF274* KO HEK293T cells. Scale bars, 10 μm. (**D**) Volcano plot of mass spectrometry analysis showing different enrichments of biotinylated proteins after targeting ΔSCAN-ZNF274-HA or ΔKRAB-ZNF274-HA in HEK293T cells (*n* = 4 replicates per condition). Color-coding of interactors is the same as in (A). (**E**) Representative IGV browser screenshot for a *KZFP* gene cluster showing tracks for H3K9me3 ChIP-seq in WT and *ZNF274* KO HEK293T cells overexpressing doxycycline-induced ZNF274-HA, ΔSCAN-ZNF274-HA, and ΔKRAB-ZNF274-HA as well as HA ChIP-seq for the same constructs expressed in *ZNF274* KO HEK293T cells. (**F**) Co-IP experiments detected by Western blot HA-tagged ZNF274 proteins, Flag-tagged ZNF274 proteins, or KAP1 in cell lysates from HEK293T cells cotransfected with ZNF274 constructs. (**G**) Normalized Hi-C pyramid plots at 100-kb resolution showing the rescue of long-range contacts (black arrow) between *PCDH* and *KZFP* gene clusters on chromosome 5 upon overexpression of ZNF274-HA but not ΔSCAN-ZNF274-HA in *ZNF274 *KO HEK293T cells.

Next, we asked how ZNF274 ablation could trigger such alterations in chromosomal conformations. Previous affinity purification and mass spectrometry experiments identified some nucleolar proteins in ZNF274 interactome, which supported earlier studies linking ZNF274 to this nuclear subcompartment (*17*, *33*). We performed proximity biotinylation experiments targeting the TurboID enzyme fused to Protein A (ProtA-TurboID) to HA-tagged ZNF274 with an HA-specific antibody, followed by streptavidin-based affinity enrichment and quantitative mass spectrometry (Fig. 4A and table S4) (*34*). Besides KAP1 and two other tripartite motif-containing (TRIM) proteins (TRIM24 and TRIM33) also part of the transcriptional intermediary factor 1 (TIF1) family, hits included several chromatin-associated proteins such as the CHD4/NuRD complex, validating our experimental approach. Sixteen of 127 significant ZNF274 interactors were proteins with known nucleolar localization, an enrichment that has never been detected in the interactome of other KZFPs. ZNF274 contains the auxiliary SCAN domain in between two repressive KRAB motifs (Fig. 4B). To characterize the specificity of ZNF274 interactions, we overexpressed mutated versions of ZNF274 bearing deletions either of the two KRAB domains (ΔKRAB-ZNF274) or the SCAN domain (ΔSCAN-ZNF274) in KO cells (Fig. 4B). Confocal imaging of full-length ZNF274 revealed its accumulation in discrete nuclear dots and frequent localization in the compartment surrounding the nucleolus (Fig. 4C); ΔKRAB-ZNF274 molecules were more prominently concentrated at nucleoli, while the SCAN-devoid derivative was excluded from these nuclear subcompartments (Fig. 4C). ProtA-TurboID experiments using ΔSCAN-ZNF274 or ΔKRAB-ZNF274 mutants as baits confirmed two markedly distinct interactomes, with nucleolus-associated proteins enriched in ΔKRAB-ZNF274 extracts and depleted in their ΔSCAN-ZNF274 counterparts (Fig. 4D and table S5). Hence, protein interactions mediated by the SCAN domain are important to favor ZNF274 nucleolar anchoring. Overexpression of full-length ZNF274 in KO cells was sufficient to restore H3K9me3 formation over target DNA, whereas ΔKRAB-ZNF274 was ineffective (Fig. 4E and fig. S10, A and B). Complementation of KO cells with ΔSCAN-ZNF274 led to a partial rescue, with some nucleation of H3K9me3 occurring on ZNF274 peaks but a defect in spreading of this histone mark. We confirmed by HA ChIP-seq that both ΔKRAB-ZNF274 and ΔSCAN-ZNF274 constructs retained the ability to bind DNA, although the average height of the peaks was somehow reduced with respect to full-length ZNF274 (fig. S10C). Of note, neither mutant induced any relevant ectopic DNA binding (fig. S10D). We recorded similar defects in repression using the GFP reporter assay, where the silencing capacity of ZNF274 was in part reduced by the absence of the SCAN domain (fig. S10, E and F). Compared to punctual nucleation, spreading of heterochromatin requires the ability of transcription factors to stabilize their contacts via homo- or heterodimerization, a known attribute of the SCAN domain (*35*). Among over 70 SCAN-containing proteins encoded in the human genome (*36*, *37*), only ZNF24 was enriched in the ZNF274 interactome (Fig. 4A). Still, neither ChIP-seq signals nor cellular localization of ZNF24 suggests a colocalization with ZNF274 (*37*, *38*). We postulated that ZNF274 would function as a homodimer accumulating around nucleoli and bringing bound loci in spatial proximity. We performed coimmunoprecipitation (co-IP) experiments by coexpressing HA- and Flag-tagged derivatives of ZNF274, which confirmed that the protein can homodimerize (Fig. 4F). Removal of the SCAN domain abolished this property, whereas the KRAB-deleted mutant failed to recruit KAP1 but maintained its dimerization capacity. Last, using the Hi-C technique, we investigated if the expression of ZNF274 or ΔSCAN-ZNF274 constructs could reestablish long-range chromosomal contacts in KO cells (fig. S6C). Although ectopic reintroduction of ZNF274 was not sufficient to reinstall genome-wide long-range plaid patterns as in WT cells (fig. S11, A and B), we could detect the reinstatement of some interactions between the *PCDH* locus and some *KZFP* genes ~10 Mb distant (Fig. 4G and fig. S11C). On the contrary, expression of the ΔSCAN-ZNF274 mutant could not foster such contact formation, even if the H3K9me3 profiles looked analogous under the two conditions (fig. S11, D and E). In conclusion, our results demonstrate that SCAN-mediated interactions promote the preferential accumulation of ZNF274 molecules in the perinucleolar environment, which in turn contributes to the segregation of lineage-specific gene clusters into repressed compartments. The fact that long-range contacts lost upon ZNF274 deletion are only minimally recovered when the KZFP is reintroduced implies that other factors may serve in the instruction of these compartments.

### ZNF274 tethers bound DNA to nucleoli

**Fig. 5.**
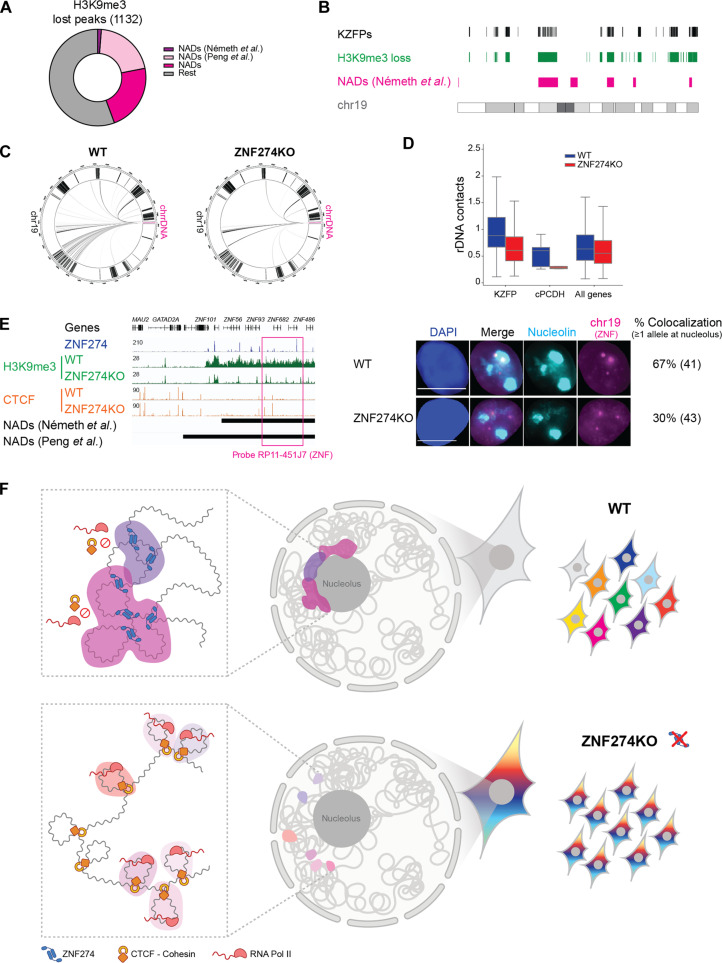
ZNF274 tethers bound DNA to nucleoli. (**A**) Doughnut chart displaying the H3K9me3-depleted regions in *ZNF274* KO cells annotated as NADs in previous publications (*8*, *9*). (**B**) Karyotype plot of chromosome 19 showing the colocalization of H3K9 hypomethylated regions in *ZNF274* KO HEK293T cells and previously annotated NADs in HeLa cells. (**C**) Circos plot representation of rDNA contacts with reads mapped on chromosome 19 in WT and *ZNF274* KO HEK293T cells using Hi-C matrixes at 100-kb resolution. Black straight lines in the outer circle represent *KZFP* genes. (**D**) Box plots (center line, median; box limits, the 25th and 75th percentiles) of *trans* interactions between rDNA sequences and the indicated set of genes [*KZFP*, *PCDH*, and all protein-coding genes (~20,000)] in WT and *ZNF274* KO HEK293T cells. (**E**) Left: IGV browser screenshot showing the localization of the probe used for DNA-FISH. Right: 2D “maximum projection” example images from immunofluorescence for nucleolin (light blue) combined with the corresponding DNA-FISH for the ZNF cluster on chr19 (pink) and DAPI, with quantification of the number of cells displaying at least one DNA-FISH probe signal contacting the nucleolus in WT and *ZNF274* KO HEK293T cells. (**F**) Model. (Top) Nucleolar tethering of ZNF274-bound clusters is functional to establish silencing of extended genomic regions and segregate them in repressive domains (dark pink) that modulate the access of CTCF and cohesin complexes to enable selective gene expression. (Bottom) Ablation of ZNF274 triggers loss of repressive chromatin marks, de novo CTCF binding, and altered 3D spatial organization of the same genomic neighborhoods (light pink), thus precluding the cell-specific fine-tuning of isoform diversity.

Many of the gene families regulated by ZNF274 are recognized building blocks of NADs (*8*, *9*). We found that about 44% (502/1132) of regions H3K9 hypomethylated in KO HEK293T cells coincide with NAD-associated sequences formerly identified in HeLa cells (Fig. 5, A and B); in comparison, only 30% (337/1132) of them correspond to LADs (*39*). Considering that NADs span a far smaller portion of the genome than LADs, a hypothesis raised by the above results is that ZNF274 might preferentially organize repressive hubs close to nucleoli. To probe our hypothesis, we recovered reads from our Hi-C maps containing contacts with rDNA, which is a major nucleolar component. Comparative analysis of interchromosomal interactions revealed that contacts between rDNA and ZNF274-targeted DNA were more frequent in WT than KO cells (Fig. 5C and fig. S12, A and B). Quantification of rDNA contacts with *KZFP* or *PCDH* genes confirmed a reduced frequency of interactions under the KO condition (Fig. 5D). To ascertain directly if ZNF274 links chromatin to the perinucleolar environment, we performed DNA fluorescence in situ hybridization (FISH) combined with immunofluorescence for nucleolin. Using a probe spanning one ZNF274-regulated *KZFP* cluster, we confirmed that at least one allele (of the three present in the triploid HEK293T cells) frequently contacts the nucleolus in WT cells (Fig. 5E). This association was reduced in KO cells, whereas nucleolar localization of the ZNF274-unbound 5*S* rDNA control region on chromosome 1 stayed unaltered (fig. S12C). These results confirm that ZNF274 has a key role in instating a comprehensive genome compartmentalization by positioning bound DNA most often near the perinucleolar environment to regulate accessibility and prevent unintended activation of gene clusters with lineage-specific functions (Fig. 5F).

## DISCUSSION

The nucleolus is a central component of nuclear architecture; yet, the molecular mechanisms behind this organization have remained elusive thus far. Here, we uncovered a previously undescribed mechanism of epigenome regulation for tandemly arrayed genes. Our data suggest a model whereby ZNF274, by targeting specific chromosomal regions near the perinucleolar environment, acts as a guardian against the uncontrolled activation of gene family clusters instead of selective promoter choices (Fig. 5F). This is reminiscent of a superrepression model proposed by Zuckerkandl in 1997, where genomic elements sharing analogous molecular regulatory features meet in the 3D space to spread chromatin modifications in *trans* (*40*). It follows that the establishment of sectorial repression must occur prior to lineage-specific transcriptional choices and its long-term stability be ensured by the constitutive production of ZNF274. Supporting this model, ZNF274 is expressed in early development starting at the four-cell stage but not in oocytes, when nucleoli transform into nucleolus precursor bodies (*41*).

The *ZNF274* gene is also intolerant to loss-of-function mutations, with only a small number of heterozygous individuals (~20) having been found in large-scale genetic surveys (https://gnomad.broadinstitute.org/). In mice, homozygous mutants of the *ZNF274* ortholog *Zfp110*/*Nrif1* on a C57BL/6NJCrl background die at embryonic day 12 (*42*). On the basis of the data presented here, it is reasonable to speculate that loss of ZNF274 is very damaging for human development or even likely embryonic lethal. ZNF274 is a known epigenetic regulator of the PWS critical region, whose abnormal DNA methylation leads to a complex neurobehavioral disorder. Besides, we identified a potential association between ZNF274, the regulation of *PCDH*, and schizophrenia pathogenesis. Connections with PWS and schizophrenia pathogenesis suggest that severe intellectual impairments may arise from the dysregulation of ZNF274 expression. Thus, the implications of our findings extend beyond the mechanisms regulating spatial genome organization and hint to a biochemical link between the structural role of NADs and healthy human development.

Last, we showed that the segregation of repressed chromatin in the perinucleolar compartment depends on the concerted activity of the KRAB and the SCAN domain of ZNF274, where the former regulates the deposition of H3K9me3 and the latter promotes nucleolar aggregation. The SCAN domain is a highly conserved vertebrate-specific motif that is thought to act as a protein interaction domain mediating self-association or heteroassociation with other proteins (*43*). The human genome encodes for over 70 SCAN ZFPs; still, the function of most of them remains unknown (*35*–*37*, *44*). Our results warrant further studies asking if this protein family may have more generally evolved to modulate the spatial genome architecture and shape cell-specific regulatory networks.

## MATERIALS AND METHODS

### Cell culture

HEK293T cell lines were maintained in Dulbecco’s modified Eagle’s medium (Gibco) supplemented with 10% fetal bovine serum and 1% penicillin/streptomycin. H1 human ESC lines (WA01, WiCell) were maintained in mTeSR Plus (STEMCELL) on Matrigel (BD Biosciences) and passaged using TrypLE in single cells. NPCs were generated from ESC in STEMdiff Neural Induction Medium (STEMCELL) using the embryoid body protocol and maintained in the STEMdiff Neural Progenitor Medium (STEMCELL) under nondifferentiating conditions or cryopreserved using the STEMdiff Neural Progenitor Freezing Medium (STEMCELL). All cells tested negative for mycoplasma.

### Lentivirus production and stable gene expression

HEK293T cells overexpressing HA-tagged constructs were generated as described by de Tribolet-Hardy *et al.* (*29*). In short, cDNAs for ZNF274 constructs were codon optimized, synthesized using the GeneArt service from Thermo Fisher Scientific, and cloned into the doxycycline-inducible expression vector pTRE-3HA. Stable cell lines were generated using the lentivector transduction of HEK293T cells at a multiplicity of infection (MOI) of 5 or 10 as described on http://tronolab.epfl.ch and selected with puromycin (5 μg/ml) or hygromycin (200 μg/ml).

ZNF274 expression was induced with doxycycline (1 μg/ml) and verified via Western blot with an anti-HA antibody (1:1000; ref. 12013819001, Roche) and anti–β-actin antibody (1:1000; ref. ab20272, Abcam).

### CRISPR-Cas9 editing

*ZNF274* was deleted in HEK293T and ESC cells using pX459 CRISPR-Cas9 system (Addgene, no. 62988). Single guide RNAs (sgRNAs) upstream and downstream of *ZNF274* were cloned into a Cas9-PuroR construct. Puromycin-resistant cells were selected after transfection with the two sgRNAs to increase the probability for a direct deletion.

The sequences of the guides are as follows: for_2A, (CACCCAAGCGGCGACACACGTTGC); rev_2A, (AAACGCAACGTGTGTCGCCGCTTG); for_2B, (CACCCAAACGTCCACCCTAGTTCT); and rev_2B, (AAACAGAACTAGGGTGGACGTTTG).

For HEK293T, 400,000 cells were transfected for 24 hours using Lipofectamine 300 (Mirus Bio, ref. MIR2300) and 2 μg of sgRNAs, selected with puromycin (2 μg/ml), and single-cell sorted with flow cytometry in a 96-well plate for screening.

For ESC, 400,000 cells were transfected for 48 hours using TransIT-LT1 (Thermo Fisher Scientific, ref. L3000008) and 2.5 μg of sgRNAs, selected with puromycin (0.5 μg/ml), and plated at a low density in mTeSR Plus. Single colonies were picked and seeded in a 96-well plate for screening.

Effective KO of *ZNF274* was validated by polymerase chain reaction (PCR) on genomic DNA and reverse transcription quantitative PCR (RT-qPCR) on RNA transcribed into cDNA using the following primers: PCR on genomic DNA: (i) WT allele set 1, (ATCCAGGCCCTATATGCTGAA, GAGCGTGCCTTAGGCTGTA); (ii) WT allele set 2, (GACAAGACCATACTGGGACC, CACGCAACTTTTGGGGAAGG); and (iii) deletion, (GACAAGACCATACTGGGACC, AGGCGAGACGCTGATTGGAT); and RT-qPCR on cDNA: (i) WT allele set 1, (ATCCAGGCCCTATATGCTGAA, GAGCGTGCCTTAGGCTGTA); and (ii) WT allele set 2, (TCAGCTTTCCAAACCAGATG, TGGAATGGTGTCTTGAGGAA).

### Chromatin immunoprecipitation

ChIP was performed as described previously (*33*). Briefly, cells were cross-linked for 10 min at room temperature (RT) in phosphate-buffered saline (PBS) + 1% methanol-free formaldehyde solution, followed by quenching with 250 mM Tris (pH 8). Nuclei were sonicated with a Covaris E220 sonicatior (settings: 5% duty, 200 cycle, 140 PIP, and 20 min) to yield genomic DNA fragments with a bulk size of 200 to 500 bp. IP was performed overnight at 4°C using 5 μg of the antibody (anti-H3K9me3, Diagenode, ref. C15410056; anti-HA.11, BioLegend, ref. 901503; anti-H3K4me3, Cell Signaling, ref. 9751S; anti-H3K36me3, Diagenode, ref. C15410192; anti-CTCF, Millipore, ref. 07-729; anti-Rad21, Abcam, ref. ab993) coupled to 50 μl of Protein G Dynabeads (Invitrogen, ref. 10009D). Final DNA was purified with a QIAGEN Elute Column. Up to 10 ng of the material for both total inputs and chromatin immunoprecipitated samples were used for library preparation. After end-repair and A-tailing, Illumina IDT indexes were ligated to the samples. Libraries were size selected using Ampure XP beads (Beckman Coulter), quality checked on a Bioanalyzer DNA high sensitivity chip (Agilent), and quantified with a Qubit dsDNA HS assay. Libraries were sequenced with 75-bp paired end on the NextSeq 500 (Illumina).

### Cut&Tag

CuT&Tag was performed as described previously (*45*). For each mark, 200,000 cells were used per sample using the anti-H3K9me3 primary antibody (Active Motif, AB_2532132). A homemade purified ProtA-Tn5 protein (3XFlag-pA-Tn5-Fl, Addgene, no. 124601) was produced and coupled with mosaic end double-stranded annealed (MEDS) oligos by the Protein Production and Purification of École Polytechnique Fédérale de Lausanne (EPFL), as previously described (*46*). The purified recombinant protein was used at a final concentration of 700 ng/μl. Libraries were sequenced with 75-bp paired end on the NextSeq 500 (Illumina).

### ProtA-TurboID

ProtA-TurboID experiments were performed as described previously (*34*). A homemade purified ProtA-TurboID protein (Pk19 ProtA-Turbo plasmid) was produced by the Protein Production and Purification of EPFL. Briefly, HEK293T cells were cultured in 15-cm dishes and induced for 3 days with doxycycline (1 μg/ml). Cells were fixed in 4 ml of PBS + 4% paraformaldehyde (PFA) for 15 min at RT and washed twice with PBS. Cell pellets were incubated for 10 min on ice in a hypotonic lysis buffer [10 mM Tris (pH 7.5), 10 mM NaCl, 3 mM MgCl_2_, 0.3% NP-40, and 10% glycerol] then centrifuged and washed to isolate nuclei. Nuclei were permeabilized with 0.3% Triton X-100 for 10 min. Nonspecific-binding sites were blocked with a blocking solution [PBS + 0.3% Triton X-100 + 3% bovine serum albumin (BSA)] for 30 min on a rotation wheel at RT. Samples were then incubated with 3 μg of anti-HA antibody (Abcam, ref. ab9110) diluted in 300 μl of the blocking solution for 1 hour at RT. After washing, nuclei were incubated with 3.5 μg of the ProtA-TurboID enzyme in 300 μl of the blocking solution for 1 hour at RT. Next, samples were incubated with 300 μl of a biotin reaction buffer (5 mM MgCl_2_, 5 μM biotin, and 1 mM adenosine triphosphate in PBS) in a thermo shaker at 1000 rpm for 10 min at 37°C and lastly lysed overnight at 4°C in 300 μl of a radioimmunoprecipitation assay (RIPA) buffer [50 mM Tris (pH 7.8), 150 mM NaCl, 0.5% sodium deoxycholate, 0.1% SDS, and 1% NP-40]. The following day, samples were sonicated using a Branson LPe Sonicator (three cycles of 20 s at 30% amplitude) and decross-linked for 1 hour at 95°C after addition of SDS to a final concentration of 1%. After an additional sonication cycle, samples were centrifuged and the supernatant was incubated with 12.5 μl of Streptavidin Sepharose High Performance beads (15511301, Cytiva) on a rotation wheel for 2 hours at 4°C. Before use, streptavidin beads were chemically modified to be protease-resistant as described by Rafiee *et al.* (*47*). After streptavidin pull-down, beads were washed five times with the RIPA buffer and five times with PBS and eluted in an elution buffer [2 M urea, 10 mM dithiothreitol, and 100 mM Tris (pH 8)]. After incubation at 1500 rpm for 20 min at RT, 50 mM iodoacetamide was added and samples were incubated at 1500 rpm in the dark for 10 min at RT. Trypsin (2.5 μl) was then added to each sample, followed by incubation at 1500 rpm for 2 hours at RT. The eluates were collected, and 1 μl of fresh trypsin was added for overnight incubation at RT. The following day, peptides were acidified, purified on C18 StageTips, and subjected to liquid chromatography–mass spectrometry.

### Coimmunoprecipitations

Co-IPs were performed as described previously (*48*). HEK293T cells cultured in 10-cm dishes were transfected for 24 hours in doxycycline (1 μg/ml) with plasmids carrying HA-tagged constructs. Cells were washed three times PBS, lysed for 30 min at 4°C in 500 μl of an IP buffer [0.5% NP-40, 500 mM Tris (pH 7.4), 20 mM EDTA, 10 mM NaF, and 2 mM benzamidine], and centrifuged for 3 min at 5000 rpm. A fifth of the lysate was taken as the whole cell extract (WCE) control, and the rest was added to 25 μl of appropriate anti-Flag Sepharose beads or anti-HA magnetic beads for overnight incubation at 4°C. After washing, proteins were eluted off the beads in 50 μl of 2X loading buffer and boiled for 10 min. Fifteen microliters of IPed proteins and 10 μl of WCE were submitted to SDS–polyacrylamide gel electrophoresis and analyzed by immunoblotting using the anti-HA antibody (1:250; ref. 12013819001, Roche), anti-Flag antibody (1:500; ref.A8592, Sigma-Aldrich), and anti-Kap1 antibody (1:250; ref. ab10843, Abcam). Experiments were repeated three times, and representative blots are shown in the figures.

### GFP reporter assay

*ZNF274* KO HEK293T cells were transduced with pRLL-PGC-GFP or pRLL-SNP-GFP constructs at an MOI of 0.5, and GFP+ cells were isolated by flow cytometry. GFP+ cells were subjected to a second transduction with an MOI of 10 of pTRE-ZNF274–inducible constructs and selected for puromycin resistance. GFP+ HEK293T transduced with pTRE-ZNF274 constructs and control cells were treated or not with doxycycline, and the GFP signal was read by flow cytometry.

### Immunofluorescence

HEK293T cell lines were grown on coverslips covered with 0.01% poly-l-lysine (Sigma-Aldrich) and fixed for 15 to 20 min in a 4% methanol-free formaldehyde solution. Cells were washed three times with PBS, permeabilized with 0.5% Triton X-100 for 20 min, and blocked with 1% BSA for 30 min. Cells were incubated with the anti-HA antibody (1:1000; ref. ab9110, Abcam) and anti-B23 (1:500; ref. B0556, Sigma-Aldrich) in 1% BSA overnight at 4°C for 1 hour at RT. Samples were washed three times with PBS and incubated with Alexa Fluor 647–conjugated anti-mouse antibody (1:1000), Alexa Fluor 488–conjugated anti-rabbit antibody (1:1000), and 4′,6-diamidino-2-phenylindole (DAPI) (1:10,000) in 1% BSA for 1 hour at RT. Three final washes were performed before mounting the slides in Vectashield (Vector Laboratories). Images were acquired on a Leica SP8 confocal microscope using the oil objective HC PL APO 63x/1.40 and analyzed with the ImageJ software (version 2.9.0/1.53t).

### DNA-FISH

Two clones for the WT and *ZNF274* KO background were karyotyped prior to use for DNA-FISH using the CytoScan Array to ensure consistent ploidy.

HEK293T cells were grown on coverslips covered with 0.01% poly-l-lysine (Sigma-Aldrich). Cells were fixed for 10 min in a 3% methanol-free formaldehyde solution and permeabilized for 5 min in an ice-cold cytoskeleton buffer (CSK) buffer [10 mM Pipes, 300 mM sucrose, 100 mM NaCl, and 3 mM MgCl_2_ (pH 6.8)] supplemented with 0.5% Triton X-100 and 1 mM EGTA. After ribonuclease (RNase) treatment for 1 hour at 37°C with RNase A (100 μg/ml) and stored at −20°C or used directly for DNA-FISH.

DNA-FISH probes were obtained after nick translation of bacterial artificial chromosome constructs (RP11-45IJN and RP5-915 N17) purified using the Large-Construct Kit (Qiagen): 1 μg of purified DNA was labeled for 3 hours at 16°C with fluorescent deoxyuridine triphosphates (dUTPs) (SEEBRIGHT Orange 552 dUTP and SEEBRIGHT Green 496 dUTP, Enzo Life Sciences). Probes (100 ng) were supplemented with 5 μg of Cot-1 DNA (Invitrogen) and 10 μg of sheared salmon sperm DNA (Invitrogen). After precipitation, the probes were resuspended in deionized formamide, denatured for 7 min at 75°C, and further incubated for 15 min at 37°C. Probes were mixed with an equal volume of a 2X hybridization buffer [4X SSC, 20% dextran sulfate, and BSA (2 mg/ml)]. Next, coverslips were denatured for 10 min at 80°C in 70% formamide/2X SSC, dehydrated in 80 to 100% ethanol washes, and incubated with the hybridization mix at 37°C overnight in a humid chamber.

The following day, coverslips were washed three times for 4 min at 42°C with 50% formaldehyde/2X SSC (pH 7.2) and three times with 2X SSC. Then, immunofluorescence was performed at this step using anti-nucleolin (1:1000; ref. Ab22758, Abcam) and coverslips were mounted in Vectashield (Vector Laboratories). Images were acquired on a Leica DM5500 upright motorized microscope, using the oil objective HCX PL APO 63X/1.40 and with a z-stacks collected at 0.2-μm steps for each channel.

Images were processed by ImageJ (version 2.9.0/1.53t) and deconvolved using Huygens Remote Manager (version 3.7). We used the ImageJ plugin for StarDist to detect nuclei from individual cells based on DAPI staining (*49*). The Distance Analysis (DiAna) plugin was used to count the number of DNA-FISH foci per cell and calculate the distance between the DNA-FISH signal and the nucleolar marker immunofluorescence signal (*50*). Cells with three DNA-FISH spots for a given DNA loci were manually identified, and the number of cells with at least one contact with the nucleolus was visually confirmed.

DNA-FISH experiments have been performed twice, and the percentages displayed are computed from pooled values of biological replicates.

### Hi-C

The Hi-C libraries were prepared using Arima-HiC (Arima Genomics), following the manufacturer’s protocol. For all samples, either 1 million or 5 million cells were fixed in a 1% methanol-free formaldehyde solution for 10 min at RT and the reaction was quenched for 5 min with glycine to a final concentration of 125 mM. Libraries were sequenced with 100-bp paired end on the NovaSeq 6000 (Illumina).

### UMI-4C

UMI-4C experiments were performed as described previously (*51*). Briefly, 5 million cells were fixed in a 1% methanol-free formaldehyde solution for 10 min at RT and the reaction was quenched for 5 min with glycine to a final concentration of 125 mM.

Cells were lysed in an ice-cold lysis buffer [10 mM tris-HCl (pH 8), 10 mM NaCl, and 0.2% NP-40], chromatin was digested with 800 U Dpn II (NEB), and digested ends were marked with biotin (ref. 19524016, Life Technologies) prior to ligation.

Universal Primer 2 was used in all the reaction as the reverse primer (CAAGCAGAAGACGGCATACGA). The following upstream and downstream primers were used for PCR amplification: DS1_Peak2A_PCDH, AATGATACGGCGACCACCGAGATCTACACTCTTTCCCTACACGACGCTCTTCCGATCTTGTGTGATGCCATGTGTGTT; US1_Peak2A_PCDH, CAAGGAACACATAAAGGATTCC; DS3_Peak2A_PCDH, AATGATACGGCGACCACCGAGATCTACACTCTTTCCCTACACGACGCTCTTCCGATCTGTGATGCCATGTGTGTTTTTAAC; US3_Peak2A_PCDH, AGCCCACTGTGAGTTCTTACAT; DS1_Peak1_PCDH, AATGATACGGCGACCACCGAGATCTACACTCTTTCCCTACACGACGCTCTTCCGATCTTATCCACTACTGGTCATCTCA; US1_Peak1_PCDH, CATCTATTGTGTTTTTCACAGTATG; DS2_Peak1_PCDH, AATGATACGGCGACCACCGAGATCTACACTCTTTCCCTACACGACGCTCTTCCGATCTACTTATACATTTGTTCTGTT;  US2_Peak1_PCDH,  AGAGCCAGAAACTATTATTGTA; DS1_HS5-1, AATGATACGGCGACCACCGAGATCTACACTCTTTCCCTACACGACGCTCTTCCGATCTCATTCCCCGTTGTTTTGGCG; US1_HS5-1, GTGGGTTAAATTAAGCCGAGTT; DS2_HS5-1,  AATGATACGGCGACCACCGAGATCTACACTCTTTCCCTACACGACGCTCTTCCGATCTCTGAAAAATGCAGTCGACTCGC;  US2_HS5-1,  TTAATTTCCCAGGGGCAGAG; DS1_HS16-20, AATGATACGGCGACCACCGAGATCTACACTCTTTCCCTACACGACGCTCTTCCGATCTTTGTGAGGTCAGAGACTAGGGA; US1_HS16-20, TGCATTTTTCTTTTCGGCCAC; DS2_HS16-20, AATGATACGGCGACCACCGAGATCTACACTCTTTCCCTACACGACGCTCTTCCGATCTACCGCCATCTCTGTTACTGA; and US2_HS16-20, GTGGAAGCCAGGAAAGGGTG.

Libraries were sequenced with 75-bp paired end on the NextSeq 500 (Illumina).

### Enzymatic methyl sequencing

DNA (200 ng) from each cell line was processed following the NEB EM-Seq protocol (E7120S). After library preparation, the DNA was quantified and sequenced with 100-bp paired end on an Illumina NovaSeq to obtain ~100 million reads per sample.

### 10x Genomics 5′ single-cell RNA-seq

WT and *ZNF274* KO NPCs were detached using Accutase (07922, STEMCELL), washed in PBS, and immediately processed for 10x library preparation using the Single Cell 5′ v2 Gene Expression Library. All libraries were sequenced on an Illumina NovaSeq. For each condition, we aimed to collect ~4000 cells per sample.

### Data analyses

#### ChIP-seq and Cut&Tag

Raw reads were mapped to the human genome (hg19) using the short read aligner program Bowtie2, with the sensitive local mode. Before peak calling, bams were filtered to remove low-quality reads (mapq < 10) and then run through the Bioconductor package GreyListChIP (R package version 1.32.0) to mask artifact regions. For ChIP-seq, peaks were called with MACS2 using the–bampe option for the transcription factors and EPIC was used for the histone marks (*52*, *53*). High-quality peaks were kept via thresholding for score > 50 for MACS2 and false discovery rate (FDR) < 0.01 for EPIC and merged using the R package DiffBind so that only peaks that occur in at least two peak sets will be kept and merged into consensus peaks. For Cut&Tag, peak calling was done using SEACR with stringent parameters (*54*). ChIP-seq–derived motifs were found using the online RSAT tool with default parameters. BEDtools was used to perform overlap and genomic analysis alongside the R package ChIPseeker for annotation and deepTools for coverage plot (*55*–*58*). Differential binding analysis was done in R. After read quantification on peaks using BEDtools multicov -q 10 on merged peaks, a countable was produced and normalized for sequencing depth using the trimmed mean of the M-values (TMM) method as implemented in the limma package of Bioconductor (*59*). A moderated *t* test from the limma package was used to test significance. *P* values were corrected for multiple testing using the Benjamini-Hochberg’s method (*60*). Regions with an absolute fold change (FC) bigger than 2 and adjusted *P* smaller than 0.05 were considered significant.

#### RNA sequencing

Reads were mapped to the human (hg19) genome using hisat2 with parameters -k 5 --seed 42 -p 7 (*61*). For stranded data, the --rna-strandness RF parameters were added. Counts on genes and transposable elements (TEs) were generated using featureCounts (*62*). To avoid read assignation ambiguity between genes and TEs, a gtf file containing both was provided to featureCounts. For repetitive sequences, an in-house curated version of the Repbase database was used (fragmented long terminal repeat and internal segments belonging to a single integrant were merged), as in (*63*). Only uniquely mapped reads were used for counting on genes and TEs. Last, the features for which the total of reads across all samples was lower than the number of samples were discarded from the analysis. Normalization for sequencing depth was done for both genes and TEs using the TMM method as implemented in the limma package of Bioconductor and using the counts on genes as the library size. Differential gene expression analysis was performed using voom as it has been implemented in the limma package of Bioconductor. A gene was considered to be differentially expressed when the FC between groups was bigger than 2 and the adjusted *P* was smaller than 0.05. A moderated *t* test (as implemented in the limma package of R) was used to test significance. *P* values were corrected for multiple testing using the Benjamini-Hochberg’s method (*60*).

#### UMI-4C

UMI-4C data were preprocessed and analyzed with the R package umi4cPackage (with default parameters) using a custom genome including the rDNA sequence as an additional chromosome [National Center for Biotechnology Information (NCBI) U13369.1] (*51*). For comparing the KO to the WT, the KO sample was normalized to WT and the domainogram shows the FC in mean contact intensity between the two conditions. FCs at specific regions and *P* values were computed via a χ^2^ test, and error bars were estimated from the binomial SD.

#### Hi-C

Raw reads were processed through the HiC-Pro v 3.1.0 pipeline (*64*). Mapping parameters were kept to default to map on a custom genome including the rDNA sequence as an additional chromosome (NCBI U13369.1). The digestion parameters were set according to the restriction enzyme (GATCGATC, GANTGATC, GANTANTC, and GATCANTC) and the minimum and maximum insert sizes set to 100 and 1400, respectively. The contact maps were generated for 20 and 100 kb then normalized using the iterative correction and eigenvector decomposition (ICE) methods. Analysis was done using the R package GENOVA (*65*).

#### TurboID

Label-free quantifications (LFQs) were generated using the MaxQuant pipeline and then normalized using the variance stabilization normalization as implemented in the DEP R package (*66*, *67*). Missing values were imputed differently depending whether the data were missing at random (MAR) or not (MNAR). For MAR, the *k*-nearest neighbors approach was used with option rowmax = 0.9. For MNAR values, the imputation was performed drawing from a Gaussian distribution using the minProb option with parameter *q* = 0.1. For the contrasts between conditions, the threshold of FDR = 0.05 and log_2_ FC of 1.5 were chosen. Gene set enrichments were computed using the hyperGTest from the GOstats R package with *P* value cutoff of 0.05 (*68*).

#### Enzymatic methyl sequencing

EM-seq raw data were processed by the Msuite2 v.2.2.0 pipeline with parameters -3 -m BS and using hisat2 as the mapper on the hg19 version of the human genome with rDNA added as an extra chromosome (*69*). The resulting mean methylation calls across replicates were aggregated by regions of interest, and the 75th percentile of all mCG in the region was computed as summary statistic for final analysis. Plotting was performed in python3 using the seaborn package, and the statistical significance assessed by the Wilcoxon test using the SciPy Python package (*70*, *71*).

#### 10X scRNA-seq

10x data were processed by standard Cell Ranger (v7.0.0) pipeline on the hg19 genome (*72*). The count data were then loaded in Seurat v 4.3.0 for quality control and analysis (*73*). Cells with more than 200 features and less than 25% mitochondrial gene content were kept for further analysis. Seurat’s SCTransform was used to normalize the data and correct for mitochondrial percentage and the total number of read biases. Uniform manifold approximation and projection (UMAP) were computed using 30 principal components on the transformed data in Seurat. For estimating the number of *KZFP* and *PCDH* genes detected per cell, a gene was considered detected as soon as harboring at least one read. Then, the Seurat FindClusters function was used with a resolution of 0.2 to infer cell clusters. Two clusters were identified as outliers and correlated with high mitochondrial gene expression and were discarded from the rest of the analysis. For graphical representation, the scCustomize R package was used (*74*).
