# Supplementary material for: Clusters of lineage-specific genes are anchored by ZNF274 in repressive perinucleolar compartments
Source: Sci Adv. 2024 Sep 13;10(37):eado1662. doi: 10.1126/sciadv.ado1662 (PMC11397430; doi:10.1126/sciadv.ado1662)
Supplement: Supplementary file 1 — Figs. S1 to S12 Legends for data S1 to S6 [file sciadv.ado1662_sm.pdf]

Science Advances
AAAS

# Supplementary Materials for

## Clusters of lineage-specific genes are anchored by ZNF274 in repressive perinucleolar compartments

Martina Begnis *et al.*

Corresponding author: Martina Begnis, martina.begnis@epfl.ch; Didier Trono, didier.trono@epfl.ch

*Sci. Adv.* **10**, eado1662 (2024)
DOI: 10.1126/sciadv.ado1662

**The PDF file includes:**

Figs. S1 to S12
Legends for data S1 to S6

**Other Supplementary Material for this manuscript includes the following:**

Data S1 to S6

## SUPPLEMENTARY FIGURES:

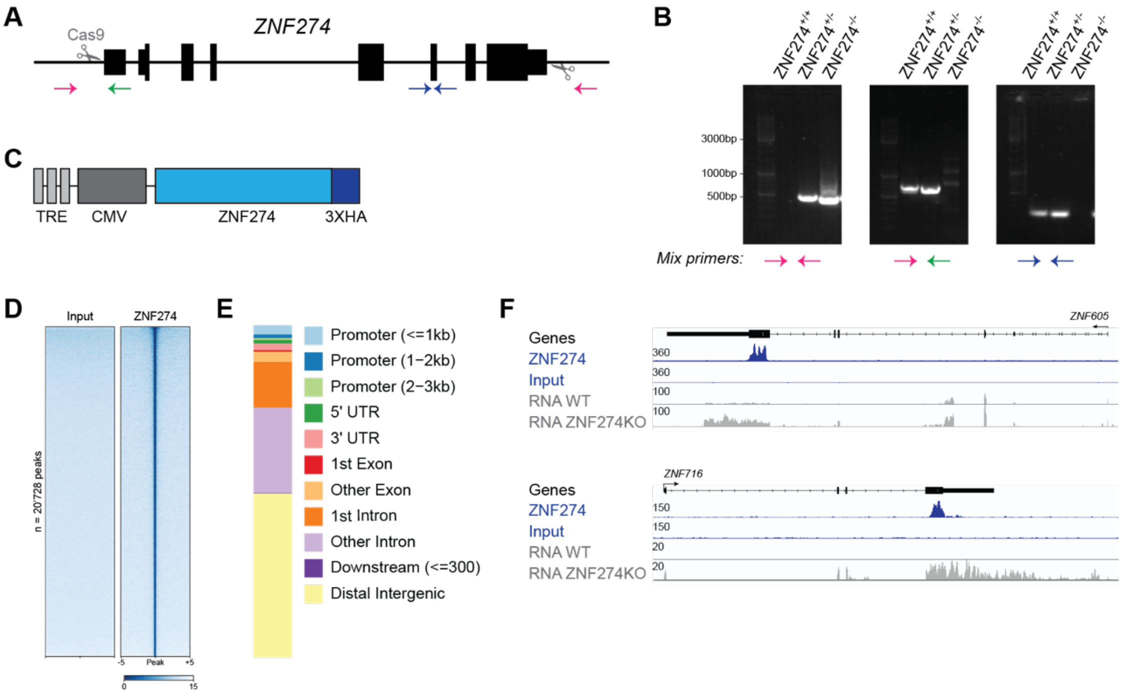


**Fig. S1.**

**(A)** Scheme depicting CRISPR–Cas9 induced double-stranded DNA breaks to delete *ZNF274* locus.

**(B)** PCR genotyping confirming homozygous deletion of *ZNF274* locus.

**(C)** Scheme depicting the construct used for doxycycline inducible ZNF274 expression system.

**(D)** Heatmaps of ZNF274-HA ChIP-seq enrichment at significant ZNF274 peaks in HEK293T. Each row represents a 5 kb window centered on peak midpoint, sorted by ZNF274 ChIP signal.

**(E)** Bar plot showing the percentage of genomic features overlapping with ZNF274 peaks.

**(F)** IGV browser screenshot of representative KZFP genes showing tracks for ZNF274 ChIP-seq in *ZNF274* KO HEK293T cells overexpressing HA-tagged ZNF274, and RNA-seq in wild-type and *ZNF274* KO HEK293T cells.

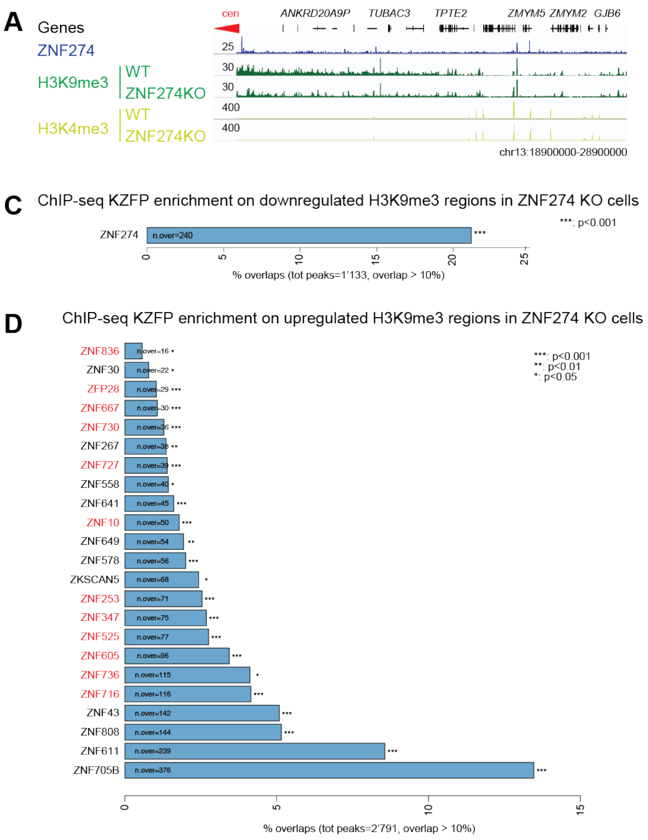


**Fig. S2.**

**(A)** IGV browser screenshots showing tracks for ZNF274 ChIP-seq in *ZNF274* KO HEK293T cells overexpressing HA-tagged ZNF274; H3K9me3 and H3K4me3 ChIP-seq in wild-type and *ZNF274* KO HEK293T cells in the pericentromeric region of chromosome 13. The red arrow indicates the position of centromeric repeats.

**(B)** Heatmap of H3K9me3 ChIP-seq enrichment across regions showing differential enrichment of H3K9me3 in wild-type versus *ZNF274* KO HEK293T cells.

**(C)** Barplot representing the overlap of significantly decreased H3K9me3 peaks with annotated peaks for KZFPs (at least 10% overlap). P-values asses the enrichment of the KZFP on those regions.

**(D)** Barplot representing the overlap of significantly increased H3K9me3 peaks with annotated ChIP-seq peaks for KZFPs (at least 10% overlap). P-values asses the enrichment of KZFPs on those regions. KZFPs in red are those whose gene expression significantly changes in wild-type versus *ZNF274* KO HEK293T cells.

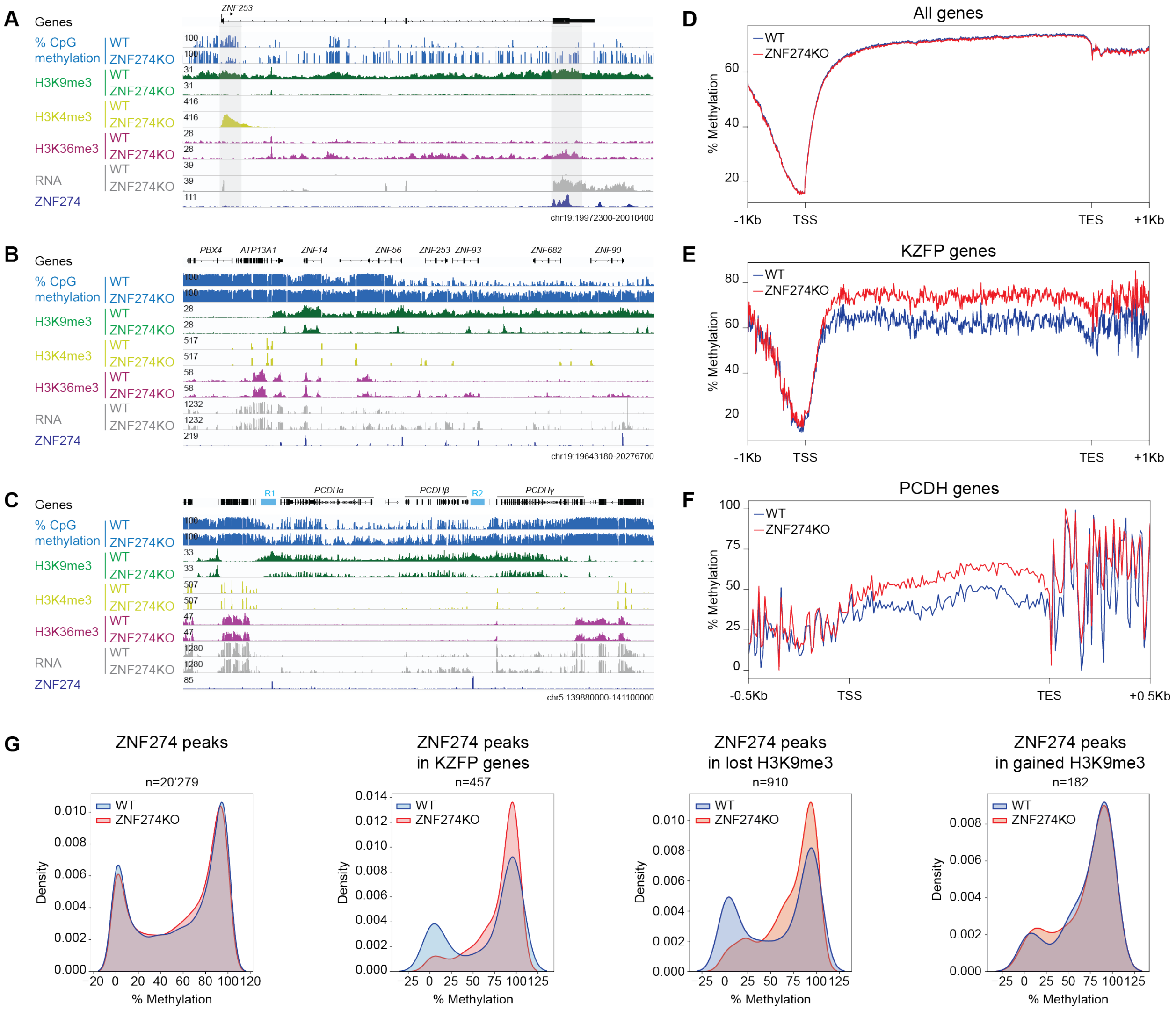


**Fig. S3.**

**(A)** IGV browser screenshot of *ZNF253* gene showing tracks for EM-seq (% CpG methylation), RNA-seq and ChIP-seq for H3K9me3, H3K4me3 and H3K36me3 and ZNF274-HA in wild-type and *ZNF274* KO HEK293T cells. The two gray boxes point to the 3' and 5' end of the gene showing changes in % of DNA methylation.

**(B)** IGV browser screenshot of *KZFP* gene cluster showing tracks for EM-seq (% CpG methylation), RNA-seq and ChIP-seq for H3K9me3, H3K4me3 and H3K36me3 and ZNF274-HA in wild-type and *ZNF274* KO HEK293T cells.

**(C)** IGV browser screenshot of *PCDH* gene cluster showing tracks for EM-seq (% CpG methylation), RNA-seq and ChIP-seq for H3K9me3, H3K4me3 and H3K36me3 and ZNF274-HA in wild-type and *ZNF274* KO HEK293T cells.

**(D)** Signal profile for DNA methylation (%) over all genes, including 500bp upstream and downstream the gene body.

**(E)** Signal profile for DNA methylation (%) over *KZFP* genes, including 500bp upstream and downstream the gene body.

**(F)** Signal profile for DNA methylation (%) over *PCDH* genes, including 500bp upstream and downstream the gene body.

**(G)** Density plots of DNA methylation levels (%) measured by EM-seq on genomic regions carrying the indicated marks.

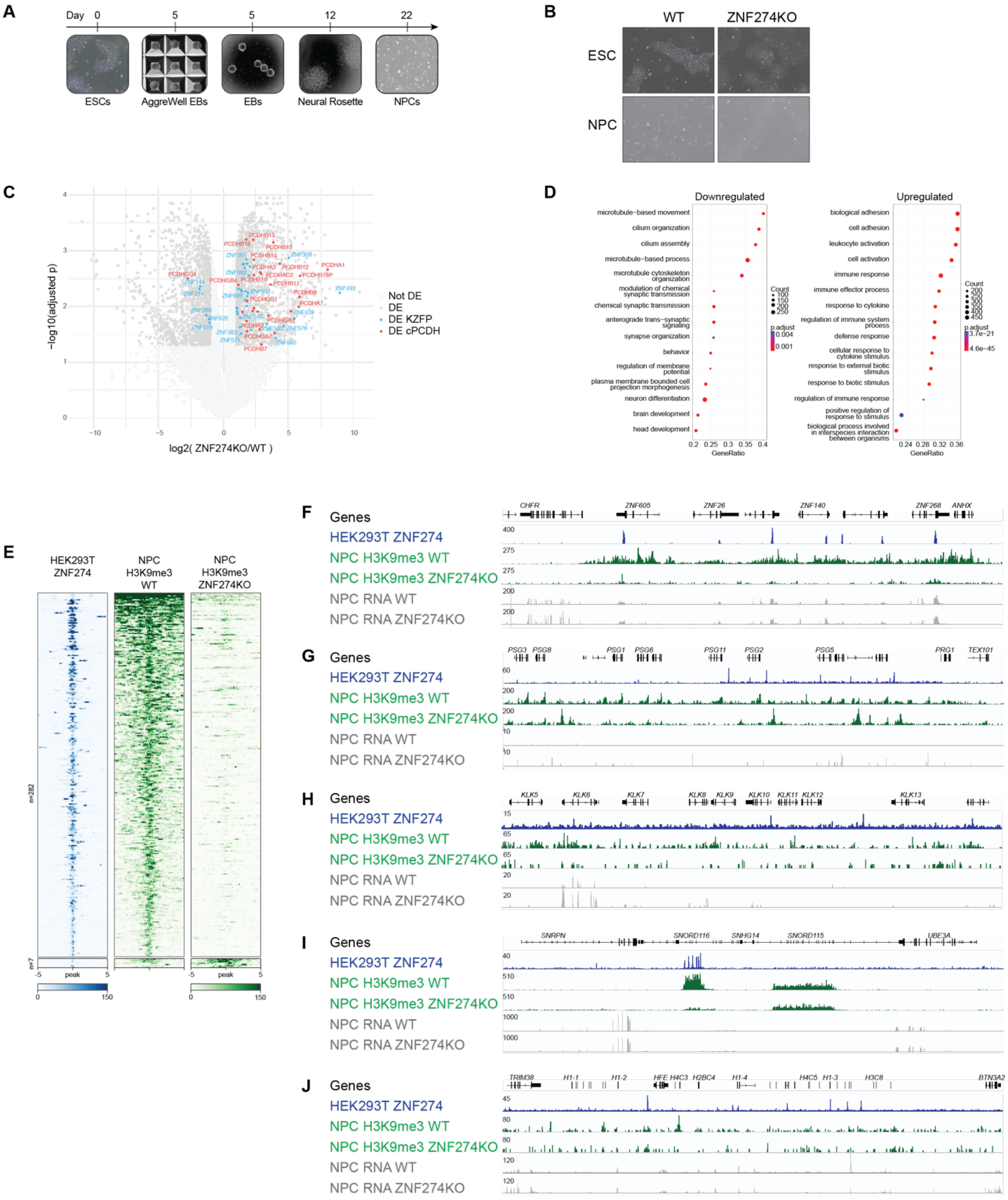

**Fig. S4.**

**(A)** Schematic of embryoid body (EB) protocol used to generate NPCs from wild-type or *ZNF274* KO ESCs.

**(B)** Phase contrast images of wild-type and *ZNF274* KO ESCs and NPCs.

**(C)** Volcano plot comparing fold change in gene expression for wild-type versus *ZNF274* KO NPCs. Representative *KZFP* and *PCDH* genes are highlighted in light blue and pink.

**(D)** Dot plots of enriched gene sets in wild-type versus *ZNF274* KO NPCs. The X-axis represents the ratio of differentially expressed genes (DEGs) in these groups over the total number of genes of each pathways (gene ratio). Dot size represent the number of DEGs in each gene set (count) and color scale represents the adjusted P value (p.adjust<0.05).

**(E)** Heatmap of ZNF274 ChIP-seq enrichment in HEK293T and differentially enriched H3K9me3 Cut&Tag peaks in wild-type versus *ZNF274* KO NPCs. Each row represents a 5 kb window centered on ZNF274 peak midpoint.

**(F)** IGV browser screenshot of a *KZFP* cluster showing tracks for ZNF274 ChIP-seq in HEK293T cells, H3K9me3 Cut&Tag and RNA-seq in wild-type and *ZNF274* KO NPCs.

**(G)** IGV browser screenshot of the pregnancy-specific glycoprotein (*PSG*) gene family showing tracks for ZNF274 ChIP-seq in HEK293T cells, H3K9me3 Cut&Tag and RNA-seq in wild-type and *ZNF274* KO NPCs.

**(H)** IGV browser screenshot of the kallikrein *(KLK)* gene family showing tracks for ZNF274 ChIP-seq in HEK293T cells, H3K9me3 Cut&Tag and RNA-seq in wild-type and *ZNF274* KO NPCs.

**(I)** IGV browser screenshot of the *SNORD116* gene cluster showing tracks for ZNF274 ChIP-seq in HEK293T cells, H3K9me3 Cut&Tag and RNA-seq in wild-type and *ZNF274* KO NPCs.

**(J)** IGV browser screenshot of the *HIST1* gene cluster on chromosome 6 showing tracks for ZNF274 ChIP-seq in HEK293T cells, H3K9me3 Cut&Tag and RNA-seq in wild-type and *ZNF274* KO NPCs.

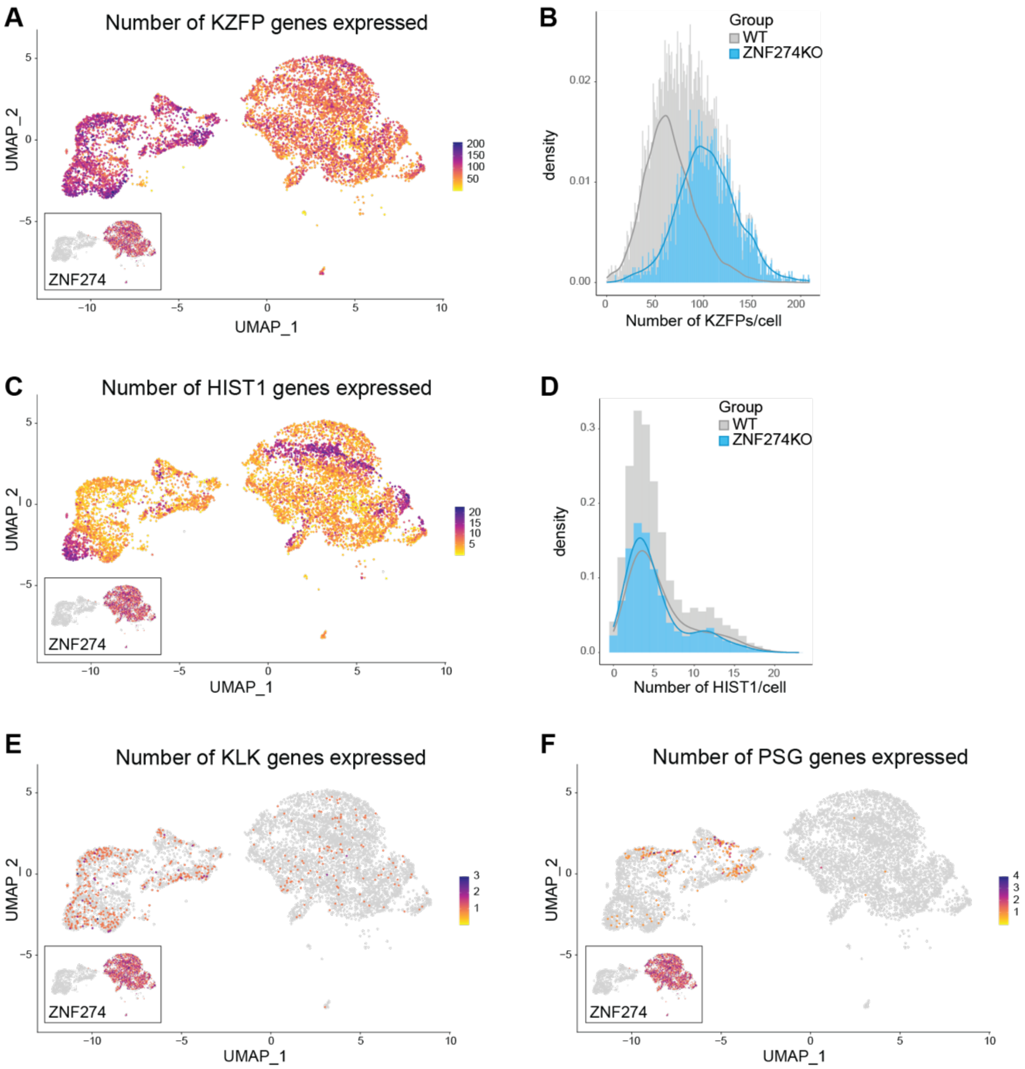


**Fig. S5.**

**(A)** UMAP visualization of wild-type and *ZNF274* KO NPCs obtained through 10X 5'-end single-cell sequencing. The UMAP is colored by colored by expression of number of *KZFP* genes. On the left bottom corner, representation of the same UMAP showing *ZNF274* expression.

**(B)** Density plot representing the distribution of cells expressing varying numbers of *KZFP* genes either in wild-type and *ZNF274* KO condition. The superimposed curves represent kernel density estimate for each condition.

**(C)** The same UMAP as in (A), colored by expression of number of *HIST1* genes from chr6 cluster.

**(D)** The same density plot as in (B) representing the distribution of cells expressing varying numbers of *HIST1* genes.

**(E)** The same UMAP as in (A), colored by expression of number of *KLK* genes from chr19 cluster.

**(F)** The same UMAP as in (A), colored by expression of number of *PSG* genes from chr19 cluster.

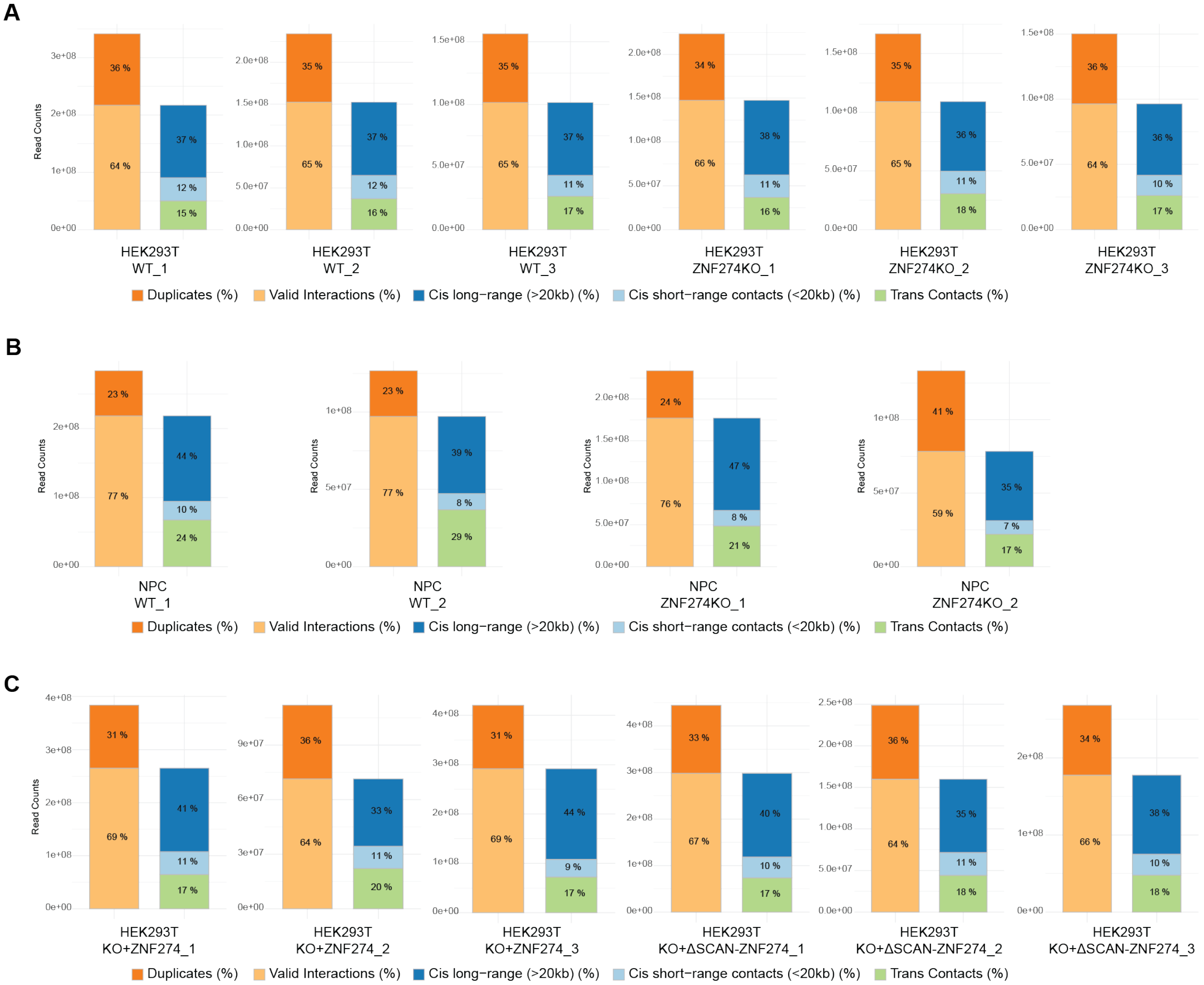


**Fig. S6.**

**(A, B and C).** Stacked barplot classifying the Hi-C valid pairs (duplicates and contact ranges) in cis short-range, cis long-range and trans contacts for all replicates separately, for the indicated cell lines.

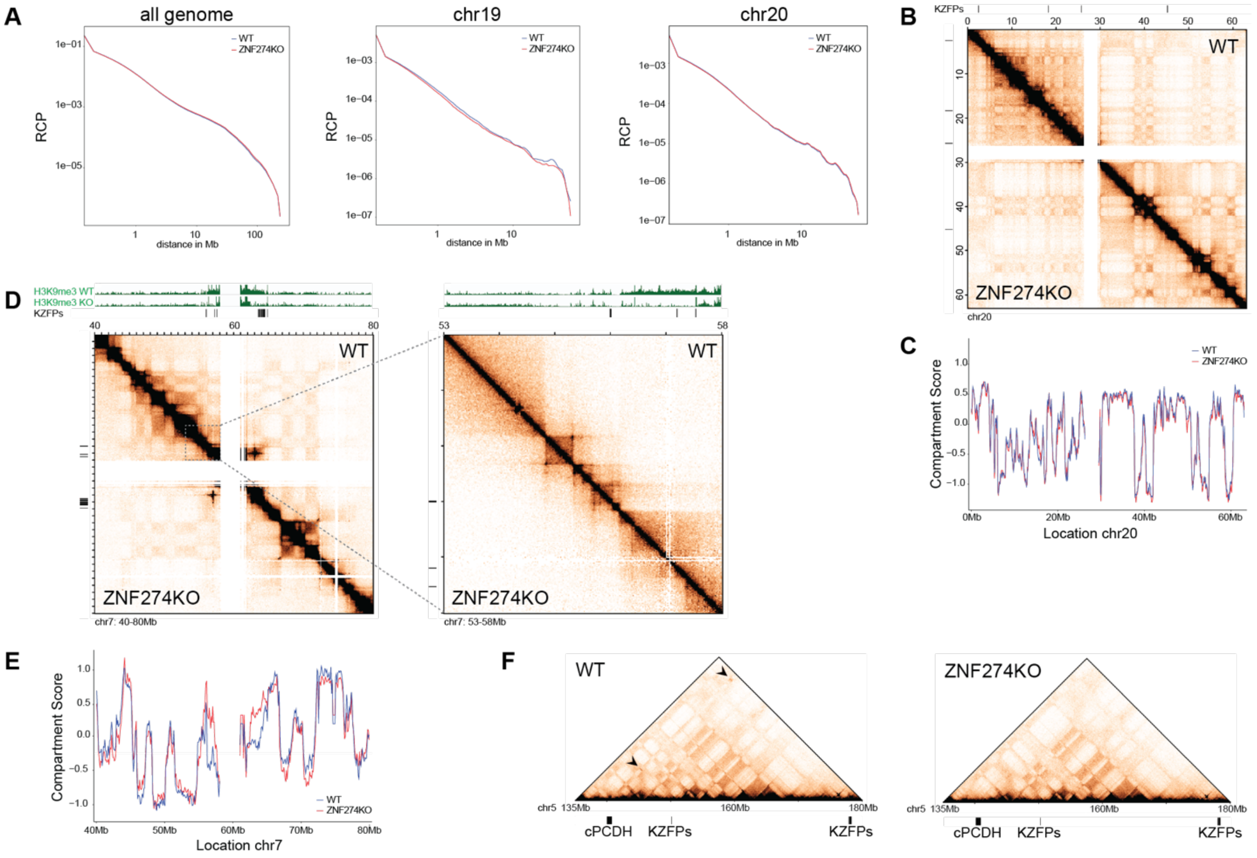


**Fig. S7.**

**(A)** Relative contact probability (RCP) plots displaying contact frequency relative to genomic distance in *cis* either for all chromosomes (left panel), chromosome 19 only (central panel) or chromosome 20 (right panel).

**(B)** Normalized Hi-C matrix at 100kb resolution of chromosome 20 displaying contact frequencies in wild-type (upper triangle) and *ZNF274* KO (lower triangle) HEK293T cells.

**(C)** Compartment scores of chromosome 20 comparing changes in segregation into A (>0) and B (<0) compartments in wild-type versus *ZNF274* KO HEK293T cells.

**(D)** Left panel: Normalized Hi-C matrix at 100kb resolution of the indicated region of chromosome 7 displaying contact frequencies in wild-type (upper triangle) and *ZNF274* KO (lower triangle) HEK293T cells. Right panels: Hi-C maps at 20kb resolution for the region highlighted.

**(E)** Compartment scores of the indicated region of chromosome 7 comparing changes in segregation into A (>0) and B (<0) compartments in wild-type versus *ZNF274* KO HEK293T cells.

**(F)** Pyramid plot at 100kb resolution showing long-range contacts (black arrows) between *PCDH* and two *KZFP* gene clusters on chromosome 5 in wild-type versus *ZNF274* KO cells.

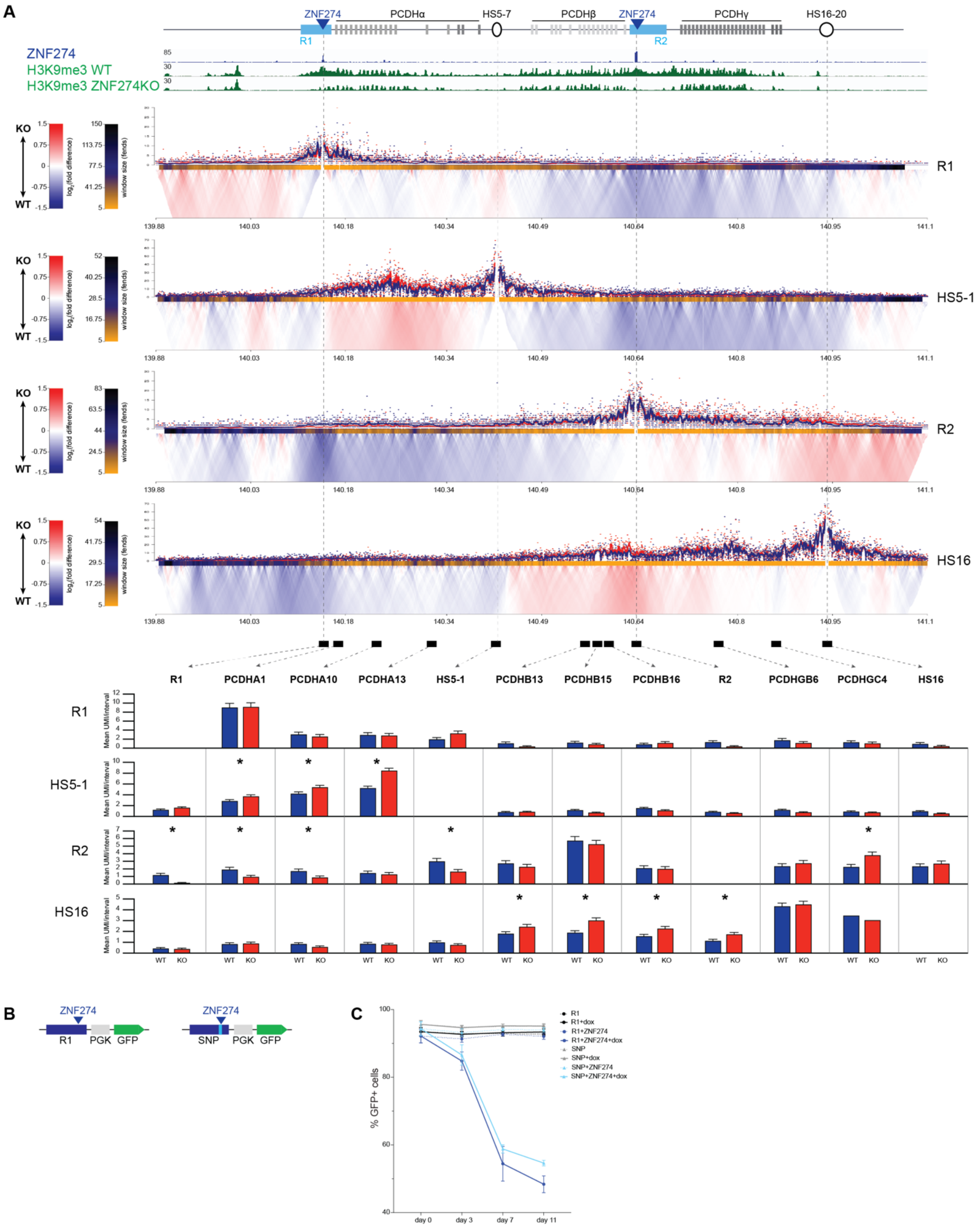


## Fig. S8.

**(A)** Upper panel: IGV browser screenshot showing tracks for ZNF274 ChIP-seq and H3K9me3 ChIP-seq on the PCDH locus. Outlined circles represent enhancer elements (HS5-7 and HS16-20); blue arrows

represent ZNF274 binding sites; light blue boxes represent R1 and R2 regions. Middle panel: Profile comparison of wild-type and *ZNF274* KO cells for four UMI-4C baits. Lower panel: Fold change of the contact intensities of wild-type and *ZNF274* KO cells over 12 genomic intervals (black boxes) along the PCDH region. Asterisk denotes $P < 0.05$ for comparison in wild-type versus *ZNF274* KO cells.

**(B)** Schematic depicting the repression assay. A 1.1 kb fragment from the R1 locus including ZNF274 binding motif +/- the polymorphism *rs111896713* (SNP) was cloned upstream the hPGK promoter.

**(C)** Silencing rate of a R1-GFP or SNP-GFP cassette when over-expressing (+dox) or not ZNF274 in transduced (+ZNF274) or untransduced *ZNF274* KO HEK293T mutants.

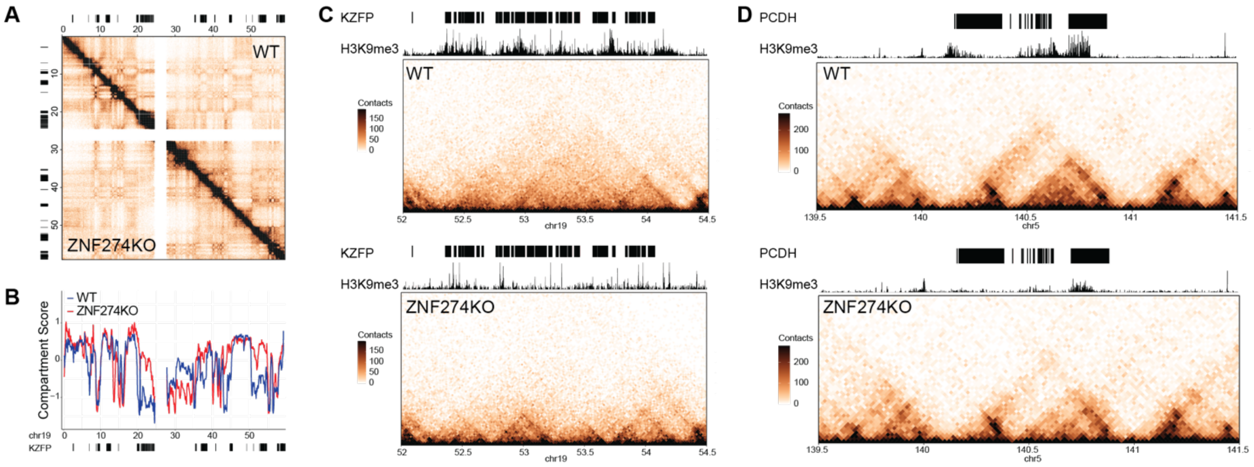


**Fig. S9.**

**(A)** Normalized Hi-C matrix at 100kb resolution of chromosome 19 displaying contact frequencies in wild-type (upper triangle) and *ZNF274* KO (lower triangle) NPCs.

**(B)** Compartment scores of chromosome 19 show changes in segregation into A (>0) and B (<0) compartments between wild-type and *ZNF274* NPCs.

**(C)** Normalized Hi-C maps at 20kb resolution for a *KZFP* gene cluster in wild-type and *ZNF274* NPCs. On the top are reported tracks visualizing *KZFP* genes (black bars) and Cut&Tag signal of H3K9me3 for each relative condition.

**(D)** Normalized Hi-C maps at 20kb resolution for the *PCDH* gene cluster in wild-type and *ZNF274* KO NPCs. On the top are reported tracks visualizing *PCDH* genes (black bars) and Cut&Tag signal of H3K9me3 for each relative condition.

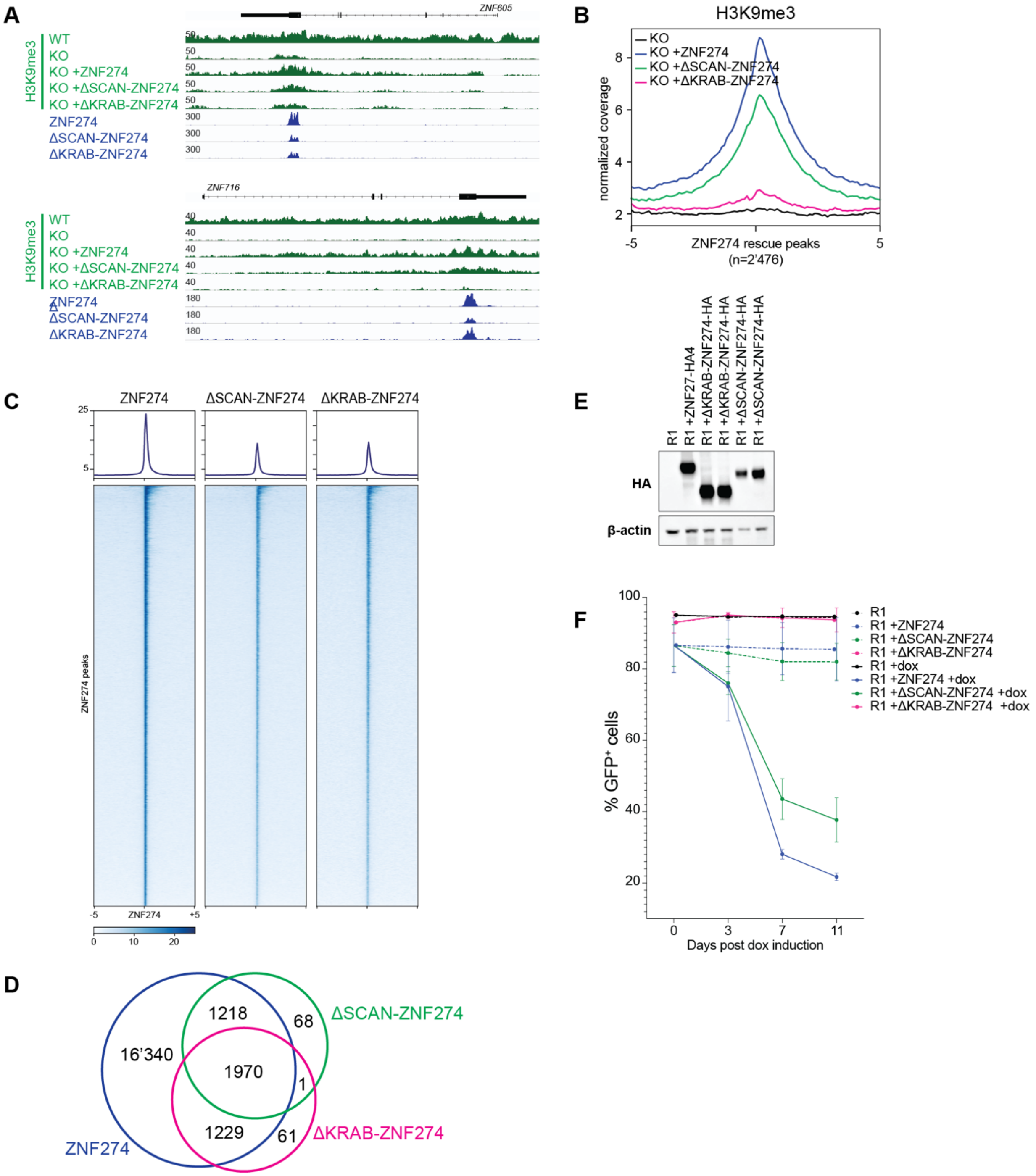


**Fig. S10.**

(**A**) IGV browser screenshots of two *KZFP* genes (*ZNF605* and *ZNF716*) showing tracks for HA ChIP-seq and H3K9me3 ChIP-seq in wild-type or *ZNF274* KO cells overexpressing doxycycline induced ZNF274-HA, ΔKRAB-ZNF274-HA, ΔSCAN-ZNF274-HA.

(**B**) Signal profile for ChIP-seq enrichment across all ZNF274 peaks where we could detect rescue of H3K9me3 deposition in *ZNF274* KO cells overexpressing ZNF274-HA.

**(C)** Heatmaps of ZNF274-HA, ΔSCAN-ZNF274-HA and ΔKRAB-ZNF274-HA ChIP-seq enrichment at significant ZNF274 peaks in *ZNF274* KO HEK293T cells. Each row represents a 5 kb window centered on peak midpoint, sorted by ZNF274 ChIP signal.

**(D)** Venn diagram showing overlap of ZNF274-HA, ΔSCAN-ZNF274-HA and ΔKRAB-ZNF274-HA ChIP-seq peaks.

**(E)** Western blot of doxycycline-induced HA-tagged constructs and beta-actin as a loading control

**(F)** Silencing rate of a PGK-GFP or SNP-GFP cassette when over-expressing (+dox) different ZNF274 constructs in transduced or untransduced *ZNF274* KO mutants.

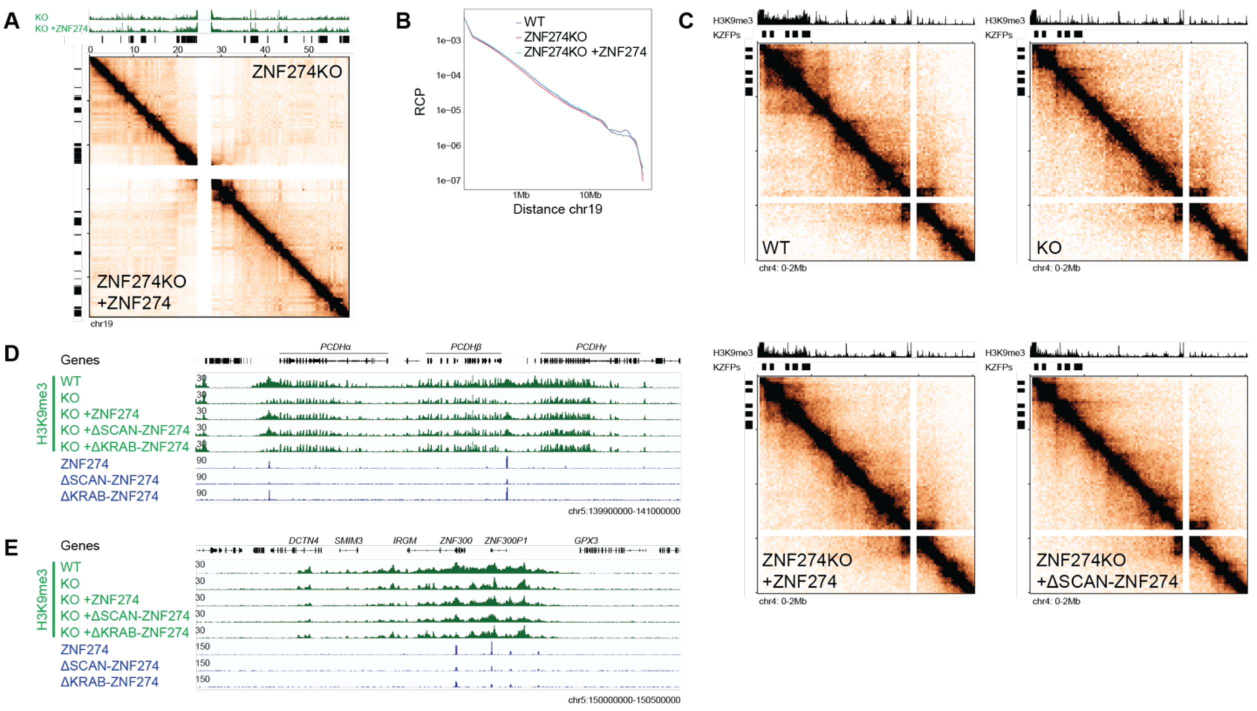


**Fig. S11.**

**(A)** Normalized Hi-C matrix at 100kb resolution of chromosome 19 displaying contact frequencies in *ZNF274* KO (upper triangle) and *ZNF274* KO +ZNF274 (lower triangle) HEK293T cells. On the top are reported tracks visualizing KZFP genes (black bars) and ChIP-seq signal of H3K9me3 for each relative condition.

**(B)** Relative contact probability (RCP) plots displaying contact frequency relative to genomic distance in *cis* for chromosome 19 in the different conditions.

**(C)** Normalized Hi-C matrixes at 20kb resolution for a *KZFP* gene cluster on chromosome 4 displaying contact frequencies in and wild-type, *ZNF274* KO, *ZNF274* KO+ZNF274 or *ZNF274* KO+ΔSCAN-ZNF274 HEK293T cells. On the top are reported tracks visualizing KZFP genes (black bars) and ChIP-seq signal of H3K9me3 for each relative condition.

**(D)** IGV browser screenshot for the *PCDH* locus showing tracks for H3K9me3 ChIP-seq in wild-type and *ZNF274* KO HEK293T cells overexpressing doxycycline induced ZNF274-HA, ΔSCAN-ZNF274-HA, ΔKRAB-ZNF274-HA, as well as HA ChIP-seq for the constructs expressed in *ZNF274* KO HEK293T cells.

**(E)** IGV browser screenshot for the *KZFP* locus forming contacts with *PCDH* which shows tracks for H3K9me3 ChIP-seq in wild-type and *ZNF274* KO HEK293T cells overexpressing doxycycline induced ZNF274-HA, ΔSCAN-ZNF274-HA, ΔKRAB-ZNF274-HA, as well as HA ChIP-seq for the constructs expressed in *ZNF274* KO HEK293T cells.

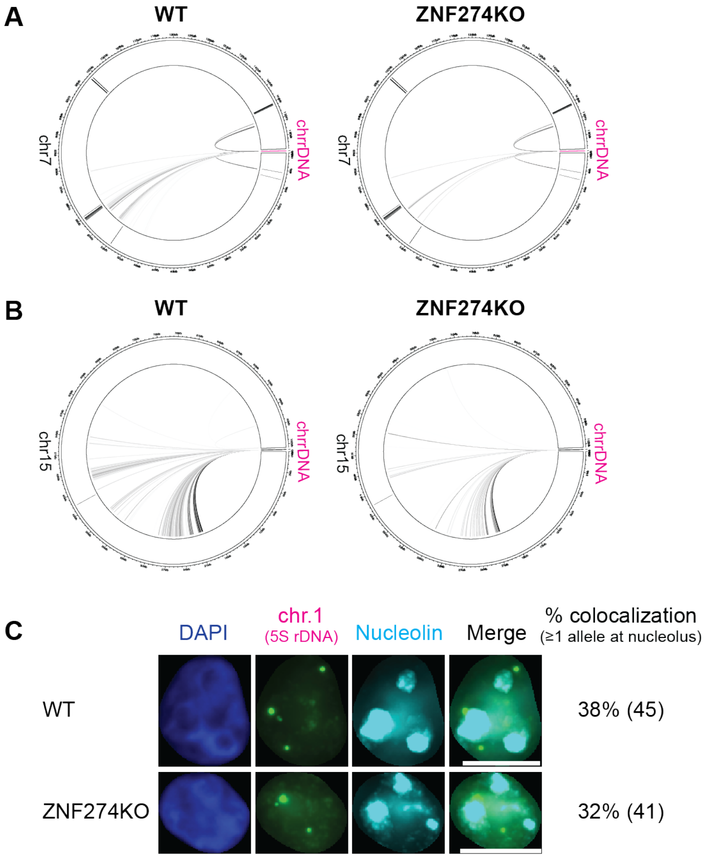


**Fig. S12.**

**(A)** Circos plot representation of rDNA contacts with reads mapped on chromosome 7 in wild-type and *ZNF274* KO HEK293T cells. Black straight lines in the outer circle represent *KZFP* genes.

**(B)** Circos plot representation of rDNA contacts with reads mapped on chromosome 15 in wild-type and *ZNF274* KO HEK293T cells. SNORD116 gene cluster lies in the 15q11-13 region located ~25 Mb where loss of contacts is detected .

**(C)** Example images from immunofluorescences for nucleolin (light blue) combined with the corresponding DNA-FISH for 5S rDNA on chromosome 1 (green) and DAPI, with quantification of the number of cells displaying at least one DNA-FISH probe signal contacting the nucleolus in wild-type and *ZNF274* KO HEK293T cells.

## SUPPLEMENTARY DATA:

### Data S1. (separate file)

The table contains averaged expression values for wild-type and *ZNF274* KO HEK293T cells, with relative fold change and associated adjusted p-value.

### Data S2. (separate file)

The table contains ZNF274 ChIP-seq peak annotations.

### Data S3. (separate file)

The table contains averaged expression values for wild-type and *ZNF274* KO NPC cells, with relative fold change and associated adjusted p-value.

### Data S4. (separate file)

The table contains relevant protein–protein interactions for HA-directed ProtA-TurboID experiment in wild-type HEK293T cell lines overexpressing HA-tagged ZNF274 versus negative untagged control.

### Data S5. (separate file)

The table contains relevant protein–protein interactions for HA-directed ProtA-TurboID experiment in *ZNF274KO* HEK293T cell lines overexpressing HA-tagged ΔSCAN-ZNF274 versus HA-tagged ΔKRAB-ZNF274.

### Data S6. (separate file)

Real-time PCR primer sequences for RAD21 ChIP.
